# Strontium Functionalization of Biomaterials for Bone Tissue Engineering Purposes: A Biological Point of View

**DOI:** 10.3390/ma15051724

**Published:** 2022-02-25

**Authors:** Giorgia Borciani, Gabriela Ciapetti, Chiara Vitale-Brovarone, Nicola Baldini

**Affiliations:** 1 Department of Biomedical and Neuromotor Sciences, University of Bologna, Via Massarenti 9, 40138 Bologna, Italy; nicola.baldini@ior.it; 2 Biomedical Science and Technologies Laboratory, IRCCS Istituto Ortopedico Rizzoli, Via di Barbiano 1/10, 40136 Bologna, Italy; gabriela.ciapetti@ior.it; 3 Laboratory for Nanobiotechnology, IRCCS Istituto Ortopedico Rizzoli, Via di Barbiano 1/10, 40136 Bologna, Italy; 4 Department of Applied Science and Technology, Politecnico di Torino, Corso Duca degli Abruzzi 24, 10129 Torino, Italy; chiara.vitalebrovarone@polito.it

**Keywords:** strontium, strontium ranelate, osteoblast, osteoclast, bone tissue engineering, scaffolds, calcium phosphate ceramics, bioactive glasses, metal-based materials, polymers

## Abstract

Strontium (Sr) is a trace element taken with nutrition and found in bone in close connection to native hydroxyapatite. Sr is involved in a dual mechanism of coupling the stimulation of bone formation with the inhibition of bone resorption, as reported in the literature. Interest in studying Sr has increased in the last decades due to the development of strontium ranelate (SrRan), an orally active agent acting as an anti-osteoporosis drug. However, the use of SrRan was subjected to some limitations starting from 2014 due to its negative side effects on the cardiac safety of patients. In this scenario, an interesting perspective for the administration of Sr is the introduction of Sr ions in biomaterials for bone tissue engineering (BTE) applications. This strategy has attracted attention thanks to its positive effects on bone formation, alongside the reduction of osteoclast activity, proven by in vitro and in vivo studies. The purpose of this review is to go through the classes of biomaterials most commonly used in BTE and functionalized with Sr, i.e., calcium phosphate ceramics, bioactive glasses, metal-based materials, and polymers. The works discussed in this review were selected as representative for each type of the above-mentioned categories, and the biological evaluation in vitro and/or in vivo was the main criterion for selection. The encouraging results collected from the in vitro and in vivo biological evaluations are outlined to highlight the potential applications of materials’ functionalization with Sr as an osteopromoting dopant in BTE.

## 1. Introduction

Strontium (Sr), a chemical element with the atomic number 38, was named after Strontian, the village in Scotland where the mineral was discovered in a mine in 1790 [1]. Sr is an abundant trace element in ocean water, ground water, and the earth’s crust, and it is introduced into the human body through nutrition. In particular, Sr may be found in the highest concentration in leafy greens (64 mg/kg), grains (18 mg/kg), and seafood (24 mg/kg) [2]. The dietary amount of Sr varies considerably without affecting human health. The assumption of Sr is not subjected to homeostatic control, and consequently the blood and serum levels are not kept constant [3].

The similarity in size and charge between Sr and calcium (Ca) allows the incorporation of Sr into the mineral phase of bone [4]. Sr and Ca are alkaline earth metals from the second column of the periodic table with two valence electrons in their highest-energy orbitals (*ns*^2^), and both form ions with a positive (+2) charge. This makes them similar in their chemical and physical properties.

However, the total amount of Sr in the skeleton is small in comparison to that of Ca since it reaches about 0.035 % of the total Ca content [5]. Even if in the past Sr has attracted less attention than other divalent metals such as Ca and magnesium (Mg), the interest regarding Sr has increased in the last decades due to the development of strontium ranelate (SrRan), an orally active agent acting as an anti-osteoporosis drug [6,7,8]. SrRan is a salt of thiopheneacetyl acid, where two atoms of stable divalent Sr are linked to an organic moiety, the ranelic acid. Sr in the form of such a salt was firstly introduced by Marie et al. in 1993 [9], while SrRan was developed as a drug by Servier (France) [10].

The first therapeutic use of stable Sr (non-radioactive) dates back to 1952, when Shorr and Carter observed that the administration of Sr lactate to osteoporotic patients, together with the administration of Ca supplements, improved the mineralizing capability of the skeleton [11]. Such a clinical study reported that osteoporotic patients had increased bone mass, reduced bone pain and enhanced mineralization following Sr administration. Subsequently, animal trials and human clinical studies showed evidence of anti-fracture efficacy and ability to increase bone-mineral density in post-menopausal women after Sr administration [7,12,13].

In light of the above, SrRan was used to treat post-menopausal osteoporosis in more than 70 countries for several years [14]. However, SrRan was withdrawn from treatment in 2014 due to the serious side effects as discussed in Section 2 of this review. 

Therefore, an alternative route for the administration of Sr has to be considered to minimize the side effects associated with oral intake. The local delivery of Sr by functionalization of the implantable biomaterials resulted in an interesting alternative [15]. Indeed, some authors reported that Sr-enriched biomaterials lead to better results than Sr-free counterparts, as observed both in vitro and in vivo: Sr (i) improves cell viability of osteogenic lineage cells, (ii) enhances the expression of osteogenic markers, (iii) stimulates new bone tissue formation when materials are implanted in vivo [16,17,18,19]. 

In light of the above, several attempts to incorporate Sr into biomaterials to locally deliver Sr ions to the area of bone where repair is required have been performed and listed in the current review. Understanding whether the local ion delivery to the bone microenvironment can be a valid alternative to the systemic administration deserves an in-depth analysis to distinguish the effects induced by surface contact from those derived from the systemic administration of Sr [20]. 

Thus, four classes of biomaterials mainly used in BTE, i.e., calcium phosphate ceramics, bioactive glasses, metal-based materials, and polymers, are considered. The functionalization with Sr ions and the in vitro and/or in vivo evaluations are briefly discussed and reported in Tables to underline the promising introduction of Sr-functionalization in treating bone defects or diseases as a potential osteopromoting dopant. 

## 2. Strontium and Strontium Ranelate

**Figure 1:**
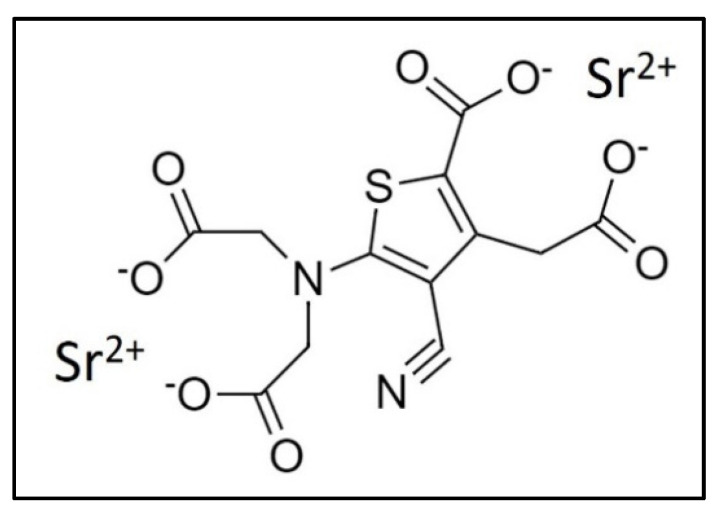
Strontium ranelate.

Strontium ranelate (SrRan) (Figure 1) has played an important role in Europe for the treatment of osteoporosis since 2004, revealing its effectiveness mainly in the treatment of postmenopausal osteoporosis in women [21].

The great success of this drug is due to its dual anabolic and anti-resorptive role and the multidirectional effects on bone tissue: SrRan stimulates bone formation and improves bone microarchitecture by enhancing the quality of bone tissue, while preventing bone loss and reducing the differentiation and resorption activity of osteoclasts [22].

Two randomized placebo-controlled phase 3 clinical studies demonstrated the effectiveness of this drug in 2004 and 2005: SOTI (Spinal Osteoporosis Therapeutic Intervention) showed the reduction in the incidence of spine fractures in postmenopausal women after just one year of treatment, while TROPOS (TReatment of Peripheral OSteoporosis) demonstrated a reduction in the incidence of non-vertebral fractures within the first three years of use [23]. 

However, SrRan was abandoned in 2014 due to its negative side effects on cardiac safety of patients, in terms of enhanced cardiovascular risk and non-fatal myocardial infarctions; consequently, the European Medicines Agency (EMA) restricted the use of SrRan introducing some limitations and publishing guidelines for the use of SrRan with several restrictions [24,25]. Patients with a history of heart or circulatory problems, such as stroke and heart attack, must not take the medicine [25]. The Agency’s Committee for Medicinal Products for Human Use (CHMP) considered that “the cardiovascular risk in patients taking Protelos/Osseor can be managed by restricting its use to patients with no history of heart and circulatory problems and limiting its use to those who cannot take other medicines approved for the treatment of osteoporosis”. These final recommendations from the CHMP come after the initial advice from the Pharmacovigilance Risk Assessment Committee (PRAC) to suspend the medicine due to its cardiovascular risk. The conclusion of the EMA was adopted a year later by the European Commission, in April 2014, with restrictions despite the positive opinion in terms of benefit-risk ratio. Today, the use of SrRan is indicated in male and female patients with severe osteoporosis, when the treatment with other anti-osteoporotic drugs cannot be applied, since it is contraindicated in patients with uncontrolled hypertension and those with a history of ischemic heart disease, peripheral arterial disease, and/or cerebrovascular disease [26]. So, the benefit-risk balance for SrRan has been judged positive in the cases abovementioned with a high facility for identifying and monitoring in routine medical practice (medical history and measurement of blood pressure) the contraindications. The regulator’s decision to maintain SrRan in the therapeutic armamentarium for osteoporosis has been welcomed by the bone community for several reasons, (i) many patients cannot take oral bisphosphonates due to contraindications or adverse effects and alternatives are essential, (ii) adherence with SrRan has been reported to be good, (iii) considering the other drugs available for anti-osteoporosis treatment, that is antiresorptive agents (bisphosphonates, Selective Estrogens Receptors Modulators (SERMs) and anti-RANKL agents) and bone-forming agents (parathyroid hormone 1–34), the mechanism of action of SrRan is unique and may attenuate concerns over the long-term suppression of bone turnover with antiresorptive drugs [27,28,29].

## 3. Calcium-Sensing Receptors and Strontium Binding

The calcium-sensing receptor (CaSR) is a class of G-protein-coupled receptors, with seven transmembrane helices, an extracellular N-terminal (ligand-binding site) and an intracellular C-terminal, with a long N-terminus, as the ligand binding site [30,31]. 

X-ray crystallography studies demonstrated that human CaSR has an extracellular domain that can bind Ca at three different sites (for more details on the structure and physiology of CaSR, see the review by Hannan et al. [32]). The CaSR binds to several physiological ligands, including Ca and Mg cations, L-aminoacids, polyamines and γ-glutamyl peptides such as glutathione [33]. The role of this receptor is to maintain Ca homeostasis in our body reacting to small variations of the Ca ion concentrations and activating the restoration of normal levels [15].

Beyond the parathyroid glands and the kidneys where it is mostly expressed, CaSR was also found in osteoblasts, osteoclasts precursors and mature osteoclasts [31]. 

Since Sr closely resembles Ca in its atomic and ionic properties, it acts as an agonist of the CaSR [34], activating CaSR in bone cells.

In addition to the pivotal role in the feedback regulation of extracellular free ionized Ca homeostasis [35], CaSR controls key cellular functions such as cell growth, differentiation and apoptosis in response to the binding of both Ca and Sr [31,36]. Even if Sr has a lower affinity than Ca for CaSR, bone cells are sensitive to Sr concentration and can activate different signaling pathways [37]. However, some authors have observed the effects of Sr stimulation on bone cells independently from CaSR: Fromigué et al. reported that SrRan enhances osteoblast replication in CaSR knockout mice and the signaling pathways activated in response to Sr both in a CaSR-dependent way or not were thoroughly investigated [38]. The authors observed that only after CaSR stimulation, the ERK1/2 phosphorylation resulted in increased osteoblasts and osteoblast apoptosis was prevented, while the Sr stimulation in the absence of CaSR induced the activation of the Akt pro-survival pathway in osteoblasts [38], as discussed below. Consequently, it cannot be excluded that different receptors, other than the CaSR, may be sensitive to Sr and implicated in the activation of distinct signaling pathways in response to Sr [39].

## 4. Incorporation of Strontium in Bone Tissue and Factors Influencing the Process

Sr has a great affinity for bone and, due to the physical and chemical similarity to Ca, the interaction and incorporation of Sr in the bone tissue are similar to what happens for Ca [15]. Sr retention occurs in three compartments: plasma and extracellular fluids, soft tissues, and skeleton. The amount of Sr unbound to serum proteins is removed from the body by urinary and fecal excretion while the bound part is retained in the above-mentioned compartments. An interesting aspect is that when Sr is administrated with Ca, a lower amount of Sr is absorbed from the intestine compared to the administration of Sr alone. This is because both Ca and Sr share the same carrier system in the intestine, which has a major affinity for Ca than Sr. 

In addition, Sr absorption in the intestine is vitamin D-dependent and decreases according to aging, food assumption and high dietary contents of Ca [40]. The physiological level of Sr in human plasma is about 0.11 and 0.31 µmol/L, even if it must be considered that the plasma concentration is dose-dependent following oral administration of Sr without affecting Ca concentration in the extracellular fluids. In the case of intravenous administration of pharmacological doses of Sr, there is a fall in plasma Ca level due to the transient fall in secreted parathyroid hormone (PTH) that induces a decrease in renal reabsorption of Ca [40]. Sr can diffuse to bone tissue penetrating the entire bone volume with a slow exchange process between blood and bone when in direct contact. The incorporation of Sr in bone tissue occurs mainly by two mechanisms: surface exchange or ionic substitution [41]. 

In humans, slow exchange of trace elements, such as Sr with Ca, is the dominant uptake mechanism during adulthood [42]. At the mineral level, Sr substitutes for Ca at random and is mainly incorporated by exchange onto the crystal surface. The rate at which Sr is incorporated into bone tissue is similar to that at which Ca is incorporated [40], and the distribution of Sr in the skeleton is directly proportional to the plasma levels [43]. The concentration of Sr in the bone tissue depends on the duration of exposure, gender and skeletal site. Boivin et al. observed that after a transient Sr administration the distribution of ions was heterogeneous in the mineralized bone, with higher concentration in newly formed bone rather than in old bone [44]. In newly formed bone only a few Sr atoms may be incorporated into the crystal by ionic substitution of Ca: even at high doses of Sr (3 mmol/day, for 13 weeks), less than 1 Ca ion in 10 can be substituted by Sr in the mineral [44]. 

Concerning the incorporation of Sr in cortical and cancellous bone, Boivin et al. found that Sr was heterogeneously distributed with three to four times more in new compact bone than in old one, and two and half more times in new cancellous bone than in old one [44], while Dahl et al. observed that Sr is preferably incorporated into trabecular bone than cortical bone [41]. Doublier et al. administered SrRan to patients with postmenopausal osteoporosis and observed that the Sr distribution in bone tissue was heterogeneous with a higher amount in trabecular bone than cortical bone, probably due to the higher surface-area-to-volume ratio or the increased rate of remodeling [45].

Sr exerts its effects not only on cell behavior but also on apatite crystals [46,47]. Indeed, crystals containing Sr ions result in more stability with regular shapes without any modification in crystal size. In addition, the incorporation of Sr into carbonate apatite improves the crystallinity and, as a consequence, Sr may stabilize hydroxyapatite crystals, hence inhibiting the resorption of the calcified matrix [44].

## 5. Effect of Strontium on Mesenchymal Stem Cells

Mesenchymal stem cells (MSCs) represent a promising and encouraging treatment in orthopedic surgery, due to their capability to differentiate towards multiple cell types of the mesenchyme alongside their low immunogenic potential [48,49]. Even if additional studies are needed to explore the safety and effectiveness of MSCs-based treatments in orthopedics, they are good candidates for regenerative purposes in BTE, and efforts have been made to test their role in bone regeneration [50]. In this scenario, the effects of Sr-functionalization of biomaterials on MSCs deserve special focus. 

Relevant effects on MSCs are exerted by Sr, including (i) stimulation of cell proliferation, (ii) enhancement of Ca deposition as mineral nodules by von Kossa stain, (iii) increased osteogenic differentiation by alkaline phosphatase (ALP) staining [51], and by upregulation of the expression of extracellular matrix (ECM)-related genes: type I collagen (COL1), bone sialoprotein (BSP) and osteocalcin (OCN) [52]. 

Sr also enhances the mRNA levels of CBFA1/RUNX2, BSP, and OCN and activates Wnt/β-catenin pathway with enhanced gene expression of key molecules, i.e., β-catenin and frizzled 8 (FZD8) [51,53,54]. Alongside the positive effects on osteogenic differentiation, Sr reduces the adipogenic differentiation of MSCs in a dose-dependent manner [53]. 

## 6. Effect of Strontium on Osteoblasts

Sr can enhance the replication of pre-osteoblastic cell lines and primary osteoblasts and promote osteogenic differentiation by up-regulation of runt-related transcriptional factor 2 (RUNX2): thanks to the similarity of Sr to Ca, Sr stimulates CaSR, activates mitogen activated protein kinase (MAPK) phosphorylation and cell signaling and enhances cell replication [55,56]. 

The positive effect of SrRan on the expression of RUNX2 and BSP was observed in bone marrow-derived stromal cells cultured for 21 days under differentiating conditions [57]. The expression of OCN, another important osteoblast marker, was increased, with consequent induction of in vitro osteogenesis [58]. In osteoblast precursors and mature osteoblasts, ALP activity, another marker of osteoblast differentiation, was stimulated, too [58]. Brennan et al. observed that the expression of RUNX2/CBFA1 in human primary osteoblasts was increased after 10 days of administration of SrRan in a dose-dependent manner, especially at 1 and 2 mM concentration; in addition, the osteoprotegerin (OPG) mRNA expression increased by around 50% and 200% within 24h of administration of 1 and 2 mM SrRan [59]. 

The osteoblast differentiation via CaSR-dependent way also involved the cyclooxygenase-2 (COX-2) pathway using prostaglandins responsible for the stimulation of osteogenic genes [60]. Alongside the osteogenic differentiation, Sr also enhances the bone matrix synthesis [61]. Fonseca observed that rat calvarial cultures exposed to SrRan showed an increased synthesis of COL1, a marker of osteoblastic function [62]. 

However, concerning the effect of SrRan on the matrix mineralization, conflicting results have been reported in the literature: in human osteoblasts, 0.1–2 mM Sr apparently exerts a positive effect after 14 days, as similarly observed also in mouse osteoblasts with 0.1–1 mM Sr, while in the case of MC3T3-E1 cell line no effect was observed [61,63]. Contrasting data are reported by Wornham et al. and Verberckmoes et al, who found that Sr at a 0.1–1 mM dose significantly inhibits the mineralization but not the collagenous nodule formation in primary rat osteoblasts [64,65]. An important effect of Sr is exerted onto the canonical Wnt signaling pathway. Following the activation of CaSR, the translocation of β-catenin into the nucleus takes place, and the Akt-signaling pathway is activated in human osteoblasts. 

At the same time, Sr decreases the expression of sclerostin with a positive effect on canonical Wnt signaling and bone formation [66]. Also, the FGF/FGF receptor (FGFR) system seems to be involved in Sr-mediated effect [67]: the stimulation of FGFR has an important role in bone growth and maturation due to the positive regulation of proliferation, differentiation and function of osteoblast-lineage cells [68]. Indeed, Caverzasio et al. demonstrated that the administration of an FGFR inhibitor to both MC3T3 cells and primary osteoblastic cells reduces the cell growth under exposure to Sr. Moreover, the authors showed that through an FGFR-mediated effect, Sr activates several intracellular pathways such as PLCγ, fibroblast growth factor receptor substrate (FRS2), Akt, ERK1/2, and p38 [69,70]. Also, the G protein coupled receptor family C group 6 member A (GPRC6A) plays an important role, confirming the observation that Sr-induced G-protein activation also happens in osteoblasts from CaSR−/− mice [39]. Indeed, GPRC6A receptors bind the same ligands as CaSR, such as Ca and Sr [71], thanks to the similar structure of both receptors. In light of the above, the GPRC6A receptor has a key role in bone remodeling since it has been reported that GPRC6A null-mice displayed osteopenia, reduced BMD, and decreased expression of osteoblast differentiation markers and osteoblast functionality [72,73].

Another important effect of SrRan on osteoblastic cells is the capability to reduce apoptosis, a harmful drawback enhanced in osteoporosis: this is possible thanks to the reduction of the level of caspase 3 and caspase 7 activity in serum, as observed by Brennan et al. following the addition of a 0.01 mM dose [59]. Hence, the overall available in vitro data describe Sr as a promoter of osteoblastogenesis [74].

## 7. Effect of Strontium on Osteoclasts

Sr addition was observed to reduce the carbonic anhydrase II and vitronectin receptor expression in osteoclasts, thereby inhibiting cell differentiation, with the osteoclast resorption activity reduced up to 66% [75]. It is known that carbonic anhydrase II plays a pivotal role as a key enzyme for bone resorption, being involved in the acidification process [76,77,78], while the vitronectin receptor (integrin αvβ3) is involved in the formation of podosomes, leading to the formation of the ruffled border during the cell attachment step [79]. 

Sr induces the reduction of the number of osteoclasts with more than three nuclei and a typical podosome belt in a dose-dependent manner, starting from a 0.1 mM concentration up to 24 mM, where an altering effect on the cytoskeleton of osteoclast-like cells till a total inhibition of osteoclast formation were observed [63,80]. 

When osteoclasts are seeded onto a mineralized matrix or an apatite collagen complex and SrRan is added, the resorption activity is inhibited [81]. Similarly, Gelinsky et al. observed reduced osteoclastic substrate resorption in vitro when osteoclasts are cultured on Sr-containing calcium phosphate bone cements [80]. 

Concerning the cytoskeleton remodeling, Sr was shown to disrupt the actin cytoskeleton organized in the sealing zone, hampering the baso-apical polarization and active resorption. The mechanisms at the base of Sr effects are partially due to the inhibition of the receptor activator of nuclear factor-kB ligand (RANKL)-induced nuclear translocation of nuclear factor-kB and activator protein-1, following the stimulation of CaSR [82].

Moreover, osteoclasts treated with Sr showed increased Ca-induced apoptosis. Since Sr closely resembles Ca in its atomic and ionic properties and both are agonists of the CaSR [83], Sr probably acts on CaSR to influence mature osteoclast apoptosis [84].

## 8. Effect of Strontium on Osteoblast-Osteoclast Crosstalk

**Figure 2:**
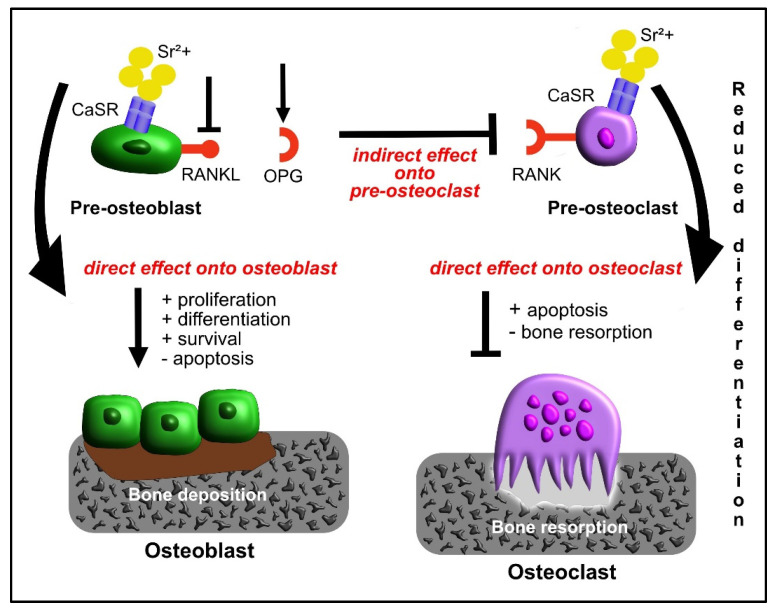
Schematic representation of the effects of Sr administration on osteoblasts and osteoclasts and their crosstalk.

As discussed above, Sr exerts its effects on osteoblasts and osteoclasts, inducing or suppressing different signaling pathways, with the ability to regulate cytokine signals between these two cell types, affecting their crosstalk. Osteoblast-lineage cells produce RANKL, a cytokine also known as osteoclast differentiation factor (ODF) that binds the cell surface receptor activator of nuclear factor-kappa B (RANK) of osteoclasts and osteoclast precursors, stimulating their differentiation. Osteoblastic cells also express OPG: this cytokine, acting as a decoy receptor, protects bone tissue from excessive resorption by blocking RANKL and preventing it from interacting with RANK, with consequent inhibition of osteoclast differentiation [85]. Consequently, the OPG/RANKL ratio is pivotal for balanced bone resorption, as proven by a decreased ratio found in bone loss circumstances, such as in post-menopausal women. Brennan et al. observed that the administration of SrRan to human primary osteoblasts, reproducing the same concentration found in the serum of patients taking 2 g per day, enhances OPG mRNA expression and OPG production and release [59]. In addition, the same authors also observed the suppression of RANKL mRNA expression and the decrease of membrane-associated protein levels of RANKL. This down-regulation of the osteoclastogenesis by indirect stimulation of osteoblasts is mediated by the activation of osteoblastic CaSR [82]. Atkins et al. pointed out that the effect of Sr on RANKL expression is not dose-dependent and may vary from donor to donor, but mRNA levels of RANKL and OPG under Sr exposure are always definitely in favor of OPG, confirming the extended inhibitory effect on osteoblast-mediated osteoclast differentiation [86]. Peng et al. observed that MC3T3 cells released OPG in the medium after treatment with Sr, and the MC3T3-conditioned medium was able to reduce the RANKL-induced pre-osteoclast differentiation and functional activity in RAW 264.7 cells [56]. This was further confirmed by the attenuation of the reduction of osteoclast differentiation following the addition of an anti-OPG antibody to the conditioned media. This evidence demonstrates the anti-catabolic effect of Sr onto osteoclasts, an effect that is mediated partly by OPG, with the indirect intervention of osteoblasts. On the other hand, Sr exerts anabolic effects onto osteoblasts, favoring the bone formation and playing an uncoupling action on bone cells, as briefly illustrated in Figure 2 [84].

## 9. Biomaterials for Bone Tissue Engineering Approach and Their Functionalization with Strontium Ions

**Figure 3:**
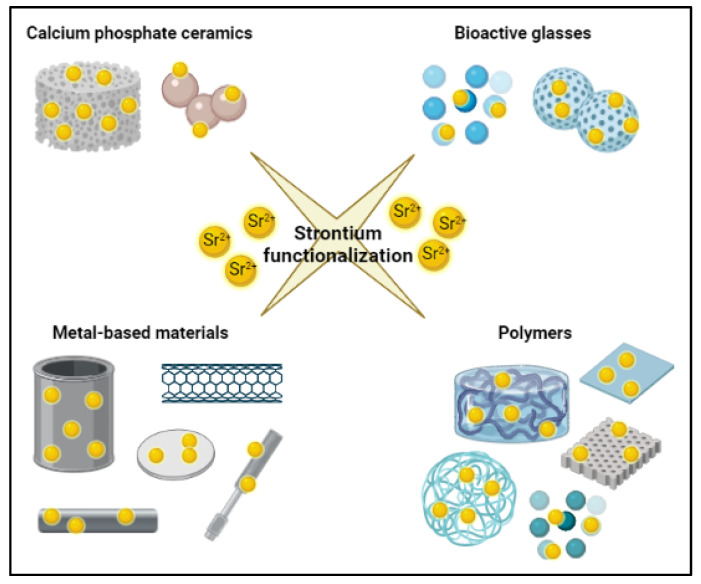
Sr-functionalized biomaterials for bone tissue engineering. The main classes of biomaterial functionalized with Sr and involved in BTE approaches are calcium phosphate ceramics, bioactive glasses, metal-based materials, polymers.

In parallel with the development of BTE, the interest in the design of biomaterials able to stimulate the regeneration of bone tissue has increased. Since the relevance of trace elements in natural bone tissue is well demonstrated, and ions such as lithium, zinc, magnesium, manganese, silicon and Sr were proven to enhance osteogenesis and neovascularization, the incorporation of dopants into biomaterials to favor bone healing was suggested by several authors [5,87,88,89,90]. In this review, we address the functionalization with Sr of the materials most commonly used for BTE, namely calcium phosphate ceramics, bioactive glasses, metal-based materials, and polymers (Figure 3). In addition, Sr-enriched biomaterials are interesting alternatives to the traditional SrRan; these smart materials allow the local delivery of Sr ions without the negative side effects that occur with the systemic administratiosn. 

### 9.1. Calcium Phosphate Ceramics

Calcium phosphate (CaP) ceramics are minerals composed of Ca cations and phosphate anions. CaP possess osteoconductive and osteoinductive characteristics and are able to dissolve in body fluids, thus exhibiting degradation and ion release [91].

These properties positively affect bioactivity in terms of cell adhesion, proliferation, and new bone formation. Ca ions of CaP enhance bone formation and maturation by (i) inducing the growth of bone cell precursors, (ii) stimulating osteoblastic bone synthesis pathway [92], (iii) increasing the life span of osteoblasts [93]. In addition, Ca ions regulate the formation and the resorptive functions of osteoclasts [94].

Alongside Ca ions effects, phosphorus ions regulate the differentiation and growth of the osteoblastic lineage and exert a negative feedback interaction between RANKL and its receptor, inhibiting osteoclast differentiation and bone resorption [95,96,97,98].

Since CaP are stiff and slow degrading materials, with structural similarity to natural bone, they are generally combined with biodegradable polymers to guarantee better structures and to enhance the mechanical performance, too low for clinical applications. The most commonly used CaP ceramics are hydroxyapatite (HA), β-tricalcium phosphate (TCP), and biphasic calcium phosphate (BCP), a mixture of the previous two materials [99]. Sr ions can be incorporated into CaP by adsorption on the surface of the mineral or, thanks to the chemical similarity between Sr and Ca, by replacing Ca with the bond to the crystalline lattice structure [15]. The considered works studying CaP functionalized with Sr are reported in Table 1. Laskus et al. reviewed the recent achievements in the field of non-apatitic CaP materials substituted with various ions, with particular attention to tricalcium phosphates and additives such as magnesium, zinc, Sr, and silicate ions [100]. HA-based scaffolds are considered an optimum material for orthopedic applications due to their chemical similarity to human bone and biocompatibility; the functionalization with Sr ions makes them more suitable for osteoporotic applications [101]. When HA-based cements are functionalized with Sr, enhanced mechanical bonding with the host tissue also under weight-bearing conditions was observed [102,103]. 

Furthermore, Sr-doped CaPS stimulated new bone growth in vivo up to 10% when implanted in segmental defects of rabbit foreleg radius [104]. 

In the work of Thormann et al, a critical-size metaphyseal defect in the femur of ovariectomized rats was filled with a Sr-modified calcium phosphate cement (SrCPC) [105]. The SrCPC, when compared to Sr-free counterpart or empty defects, showed a higher new bone formation both at the biomaterial-bone interface and in the entire fracture defect area. Using flight secondary ion mass spectrometry (TOF-SIMS) the authors detected high count rates Sr for SrCPC both in the interface region and up to a distance of 6mm to the implant, demonstrating that the enhanced new bone formation is due to local release from the SrCPC. In addition, an increased expression of ALP, OCN and type X collagen (COL10) alongside the reduction of RANKL expression were detected in the SrCPC group. 

Another important ability of calcium phosphate materials doped with Sr is the stimulation of endothelial cell proliferation and tubule formation, a desirable angiogenic ability useful for the regenerative potential [106]

Synthesized HA coatings with different proportions of Sr substitution for Ca (1, 3, or 7%) by pulsed-laser deposition were challenged with human osteoblast-like cells and osteoclasts. An enhanced osteoblast activity and differentiation alongside the inhibition of osteoclast differentiation were recorded for the highest concentration of Sr, with potentially positive effects in vivo, such as enhanced osseointegration and reduced bone resorption [107]. Interconnected porous microcarriers (Sr10–HA-g-PBLG) were prepared by grafting poly(γ-benzyl-l-glutamate) (PBLG) on Sr10–HA (Sr-doped HA) nanoparticles. The Sr10-HA-PBLG microcarriers showed a controlled-degradation rate and long-term release of Sr. Cellular evaluation with rabbit adipose-derived stem cells (ADSCs) demonstrated cell adhesion, infiltration, proliferation and the promotion of osteogenic differentiation. In vivo evaluation of bone repair potential was carried out in a critical bone defect in a rabbit model where the Sr10-HA-PBLG microcarriers seeded with ADSCs showed successful healing of the femoral bone defect [108]. Another type of Sr-HA particles was developed by Lourenço et al. by shielding the Sr-doped HA microspheres with RGD-alginate. The bone regeneration potential of the system was tested in a critical-sized metaphyseal bone defect model in Wistar Han male rats. Higher new bone formation and cell invasion were detected in the defect for the Sr-containing group compared to the Sr-free counterpart. In addition, no alteration in Sr levels in systemic organs or serum was registered [19]. Fu et al. developed a porous-core shell biphasic microspheres with 4 wt% Sr-substituted calcium silicate (CSi-Sr4) and beta-tricalcium phosphate (CaP). The effective bone regeneration process when implanted in a skull bone defect of rabbits was obtained after 12 weeks [109]. Liu et al. developed a three-dimensional (3D)-printed Sr-HA/poly(ɛ-caprolactone) (SrHA/PCL) scaffold. Rat bone marrow-derived mesenchymal stem cells (BMSCs) were seeded onto the scaffold to demonstrate that the incorporation of Sr-HA improves cell proliferation and osteogenic differentiation compared to Sr-free counterparts. Implantation of SrHA/PCL scaffold in a skull defect model in Sprague Dawley rats revealed the promotion of bone regeneration 12 weeks after implantation [110]. Porous Sr-doped (SCPC) calcium phosphate cement (CPC) scaffolds were created utilizing 3D plotting and implanted in trabecular bone defects in sheep. After 6 months, the bone formation was significantly enhanced in Sr-containing scaffold compared to Sr-free [111]. Xie et al. observed the dual effect of porous Sr-doped calcium polyphosphate scaffold (SCPP) under different Ca concentrations. When SCPP was implanted in a rabbit critical size calvarial bone, a microenvironment with a high Ca concentration, Sr accelerated bone formation, while when implanted in a subcutaneous site, considered a low Ca microenvironment, Sr inhibited bone regeneration. Thus, Sr actively participates in osteogenesis under Ca-enriched microenvironments [112]. Salamanna et al. developed a Sr-β-tricalcium phosphate (Sr-βTCP) to be tested in a spinal bone defect model in rats, to find that the implanted scaffold seeded with undifferentiated mesenchymal stem cells from bone marrow (BMSC) led to a significant spinal fusion [113]. An interesting type of calcium phosphate-based bone substitute is deproteinized bovine bone matrix (DBBM), a bone substitute of natural origin, widely used in bone augmentation procedures since it maintains the natural architecture of bone with CaP crystals similar to human bone HA [114]. Aroni et al. functionalized with Sr ions the surface of deproteinized bovine bone (Sr-DBB) to be used as implantable material for calvarial critical size defect in rats (5 mm in diameter), with two different doses of Sr loaded onto DBB particles: 19.6 μg/g and 98.1 μg/g. For both concentrations of Sr a fewer number of inflammatory cells in the bone defect site was observed and a higher amount of new bone formation was detected at 60 days when compared to Sr-free counterpart [115]. Also Elgali et al. functionalized a deproteinized bovine bone (DBB) with SrHA powders with three levels of Ca substitution by Sr: 5% (SrHA005), 25% (SrHA025) and 50% (SrHA050). Defects created in the trabecular region of rat femurs were filled with these materials and covered with a resorbable collagenous membrane. After 6 days, larger amount of bone, reduced expression of osteoclastic genes (CR and CatK) and osteoblast–osteoclast coupling gene (RANKL) were observed in the defect treated with Sr in comparison to Sr-free counterparts [116].

**Table 1:** Calcium phosphate ceramics functionalized with Sr.

Material	In Vivo/In Vitro Evaluation	Results	Reference
HA-based cements containing Sr	In vivo → goat revision hip hemi-arthroplasty model (medullary cavity of proximal femur rasped and Sr-HA cement injected)	New bone bonded to the surface of Sr-HA cement and grew along its surface	[102]
Sr-containing HA (Sr-HA) cement	In vivo → hip replacement in 12–14-months-old and 4.5–5.5 kg weighed rabbit	After 6 months from implantation, good bioactivity, stability, and bone-bonding ability under weight-bearing conditions	[103]
Porous Sr-doped calcium polyphosphate scaffolds	In vivo → implantation in segmental defects of rabbit left foreleg radius (defect size: 15 mm)	Induction of an active bone formation and extensive osteoconductivity	[104]
Sr-modified calcium phosphate cement (SrCPC)	In vivo → critical-size metaphyseal defect in the femur of ovariectomized rats	Higher new bone formation both at the biomaterial-bone interface, with increased expression of ALP, OCN and COL10	[105]
Sr-doped calcium polyphosphate (SCPP)	In vitro → endothelial cells (ECs) seeding	The surface of SCPP promotes the adhesion and spreading of ECs, improving the angiogenic behaviors	[106]
Synthesized HA coatings with different proportions of Sr substitution for Ca (0, 1, 3 or 7%)	In vitro → osteoblast-like MG-63 cells and human osteoclasts cultured on the materials	Enhanced MG-63 activity and differentiation alongside the inhibition of osteoclast differentiation	[107]
Sr-substituted HA-graft-poly(γ-benzyl-L-glutamate) hybrid nanocomposite	In vitro → cellular evaluation with rabbit adipose-derived stem cells (ADSCs) In vivo → bone repair potential in a critical bone defect in a rabbit model	In vitro → ADSCs adhesion, infiltration, proliferation, and promotion of osteogenic differentiation In vivo→ successful healing of critical bone defect in rabbits	[108]
Sr-doped HA microspheres shielded with Sr-incorporated RGD-alginate	In vivo → critical-sized metaphyseal bone defect in Wistar Han male rats	Higher new bone formation and higher cell invasion	[19]
Porous-core shell biphasic microspheres with 4 wt% Sr-substituted calcium silicate (CSi-Sr4) and beta-tricalcium phosphate (CaP)	In vivo → skull bone defect of rabbits	Bone regeneration	[109]
3D-printed Sr-HA/PCL scaffold	In vitro → rat bone marrow-derived mesenchymal stem cells (BMSCs) In vivo → implantation in Sprague Dawley rat skull defect model	In vitro → enhanced cell proliferation and osteogenic differentiation In vivo → promotion of bone regeneration after 12 weeks	[110]
Porous Sr-doped calcium phosphate cement scaffolds	In vivo → trabecular bone defects in sheep	Enhanced bone formation	[111]
Porous Sr-doped calcium polyphosphate (SCPP)	In vivo → critical size defect in rabbit calvarial bone	Sr accelerated bone formation in a highly Ca-enriched microenvironment	[112]
Sr-β-tricalcium phosphate	In vivo → scaffold seeded with undifferentiated mesenchymal stem cells from bone marrow and implanted in spinal fusion bone defect model in rats	Significant spinal fusion	[113]
Sr-loaded deproteinized bovine bone with 5%, 25% and 50% Sr	In vivo → implantation in rat calvarial critical size defect (5 mm in diameter)	A minor inflammation and a higher amount of new bone formation in bone defect site at 60 days in comparison to Sr-free counterpart	[115]
Deproteinized bovine bone functionalized with strontium-doped HA	In vivo → implantation in a bone defect in rat femoral epiphysis (trabecular bone region)	Larger amount of bone, reduced expression of osteoclastic genes (CR and CatK), and osteoblast–osteoclast coupling gene (RANKL) in the SrHA-filled defect	[116]

### 9.2. Bioactive Glasses

Bioactive glasses (BGs) are a class of synthetic inorganic biomaterials introduced in the early 1970s by Larry Hench, with the first commercialized glass named Bioglass® 45S5. BGs are widely studied for clinical applications due to their high biocompatibility and bioactivity: they easily bind to bone and soft connective tissue when implanted in vivo and release ions in the biological fluids, leading to the formation of a bone-like apatite layer on the implant surface and promoting cellular adhesion and proliferation of osteogenic cells [117,118,119]. The tunable degradation rate, the ionic release with osteogenic potential and the ability to become an HA-like material make the BGs suitable for BTE applications, even if mechanically brittle. To overcome this drawback, BGs may be combined with polymers to simulate the elastic modulus of bone better, while exhibiting increased toughness, strength, and fatigue resistance [120].

To enhance the bioactive properties, BG may be doped with ions which increase the beneficial effects for healthy bone growth [121,122,123]. In the case of BGs the level of Sr substitution modulates the amount of released strontium ions, the structure of the glass network, and consequently, the degradation and bioactivity properties [124]. Indeed, when an element is substituted by weight for another lighter element, important effects on structure and properties, as well as on biological response, may be detected. Since Sr is heavier than Ca, the substitution of 10% of the weight of Ca with Sr ions leads to less number of Sr atoms in the material [124]. 

The works studying BGs functionalized with Sr are reported in Table 2. 

As observed by Zhang et al., the incorporation of Sr ions in mesoporous BG (MBG) combines the therapeutic effects of Sr ions with the bioactivity of MBG in favor of bone regeneration. This combination can stimulate in vitro the proliferation of bone marrow-derived stromal cells and the expression of osteoblast commitment markers, i.e., ALP, COL1, RUNX2, and bone gamma-carboxyglutamate protein (BGLAP). In addition, the implantation of Sr-MBG scaffolds in critical sized femur defects in ovariectomized rats significantly stimulated new bone formation. It was observed that Sr release in blood was very low, and the excretion of Sr, Si, Ca, and P by urine was comparable to blank control animals [125]. 

Fiorilli et al. developed Sr-MBGs in the form of microspheres and nanoparticles through two different synthesis procedures, a base-catalyzed sol-gel and an aerosol-assisted spray-drying method [126]. The authors demonstrated the absence of cytotoxic effect on fibroblast cells (line L929) and the absence of inflammatory response on a murine macrophage cell line J774a.1. Moreover, the pro-osteogenic effect on osteoblast-like Saos-2 cells was shown, with the stimulation of the expression of COL1, osteonectin (SPARC - secreted protein acidic and rich in cysteine) and OPG and the downregulation of RANKL.

Pontremoli et al. modified post-synthesis Sr-MBGs by co-grafting hydrolyzable short chain silanes containing amino (aminopropylsilanetriol) and carboxylate (carboxyethylsilanetriol) moieties to achieve a zwitterion zero-charge surface [127]. The absence of cytotoxic effect on MC3T3-E1 cells, the early promotion of osteogenic differentiation and the mineral matrix deposition were observed. In addition, a significant reduction of non-specific serum protein adsorption was detected, underling the potential promotion of bone regeneration and the simultaneous inhibition of non-specific biomolecules adhesion. 

Fiorilli et al. bio-functionalized Sr-MBGs with ICOS-Fc, a recombinant molecule able to reversibly inhibit osteoclast activity by binding the respective ligand (ICOS-L), inducing the decrease of bone resorption activity, as described in a patent (WO/2016/189428) by the authors [128]. The absence of cytotoxic effect of Sr-MBGs-ICOS-Fc was evaluated on MC3T3-E1 cells. Successively, the authors confirmed the inhibitory effect of grafted ICOS-Fc on cell migratory activity by using ICOSL positive cell lines, PC-3 (prostate cancer), and U2OS (osteosarcoma). Finally, the ability to inhibit osteoclast differentiation and function was confirmed on monocyte-derived osteoclasts (MDOCs) cultured up to 21 days and exposed to Sr-MBGs-ICOS-Fc. A strong inhibition of MDOCs differentiation and a decreased formation of multinuclear tartrate-resistant acidic phosphatase (TRAP) positively stained cells were observed, together with a significant decrease in the mRNA expression of DC-STAMP, OSCAR, and NFATc1. 

In the context of silicate glass, Autefage et al. developed a 3D porous Sr-containing BG-based scaffold (pSrBG) and evaluated the absence of cytotoxic effect with MC3T3-E1 cells and the ability of bone marrow-derived human mesenchymal stem cells (hMSCs) to grow on the scaffold. Successively, the authors implanted the scaffold in a critical-sized femoral condyle defect in sheep and observed the promotion of the formation of well-organized lamellar neo-bone tissue. In particular, the Sr-containing scaffold allowed for obtaining a more mature-like lamellar bone, rather than woven bone, in comparison to the Sr-counterpart [129]. Shaltooki et al. fabricated porous nanocomposite scaffolds made of polycaprolactone (PCL) coated with a thin chitosan layer containing 15 wt% Sr-substituted BG nanoparticles (nanoparticles containing 7 wt% Sr). In vitro experiments using the MG-63 cell line showed the absence of cytotoxic effects, cell adhesion, and healthy cell morphology, as well as enhanced ALP activity in comparison to Sr-free counterpart [130]. Bellucci et al. developed bioactive glass granules combining Sr and Mg. After verifying the biocompatibility of the system with the L929 fibroblast cell line, the authors used a 3D cellular model of human bone marrow-derived mesenchymal stem cells to predict the impact of the bioactive glass granules on bone tissue. Adhesion, proliferation, and osteo-lineage differentiation were recorded [16]. Zhang et al. fabricated a temperature-sensitive p(N-isopropylacry-lamide-co-butyl methylacrylate) nanogel (PIB nanogel) scaffold functionalized with Sr-containing MBG (Sr-MBG) (1:1 mass ratio of Sr-MBG powder/PIB nanogel) to improve mechanical strength. In vitro tests with primary rat MSCs seeded onto the scaffold demonstrated an enhanced cell proliferation and ALP in the Sr-containing system compared to PIB nanogel only. The scaffold implantation into a femur defect in osteopenic rats showed that the scaffolds were able to regenerate these complicated and slow-healing critical-sized bone defects in OVX animals [131]. Different results were obtained by Poh et al. who fabricated and characterized 3D bioactive composite scaffolds of polycaprolactone (PCL) containing 45S5 Bioglass (45S5) or Sr-substituted bioactive glass (SrBG) (PCL/45S5 and PCL/SrBG). In vitro tests with MC3T3 cells showed biocompatibility and positive influence of the biomaterial on cell attachment and proliferation. However, PCL/45S5 and PCL/SrBG did not induce any difference in terms of ALP activity [132].

Wu et al. investigated whether the presence of Sr in mesoporous bioactive glass scaffold (Sr-MBG scaffold) could stimulate osteogenic/cementogenic differentiation of periodontal ligament cells (PDLCs) in a tissue-engineering scaffold system. Beyond the controlled release of Sr-MBG scaffolds, the presence of Sr significantly stimulated ALP activity and osteogenesis/cementogenesis-related gene expression of PDLCs [133]. Another example of Sr-containing mesoporous bioactive glass (Sr-MBG) scaffolds was fabricated by Zhang et al. using a 3D printing technique by preparing injectable Sr-MBG paste and adding them to 10% of PVA solution (5% or 10% or 20% of Ca was substituted with Sr). In vitro tests using MC3T3-E1 cells showed higher cell proliferation, higher ALP activity, enhanced expression of osteogenic markers RUNX2, OCN, BMP-2, COL1, and BSP, and ECM mineralized nodules formation in comparison to the Sr-free counterpart [134]. Ren et al. employing melt electrospinning developed a PCL composite scaffold incorporating 10% (weight) of Sr-substituted bioactive glass (SrBG) particles. Biological evaluation with MC3T3-E1 cells showed enhanced ALP activity, higher expression of ALP and OCN genes, and higher ECM formation compared to PCL-only scaffolds [135]. Midha et al. printed 3D hybrid constructs of silk-gelatin-bioactive glass (SF-G-BG) using two different compositions of melt-derived BGs with and without Sr. Sr-containing SF-G-BG constructs demonstrated superior osteogenic differentiation of mesenchymal stem cells, that is the up-regulation of a RUNX2, ALP, osteopontin (OPN), SPARC, BSP, and OCN expression [136]. Poh et al. developed PCL-based composite scaffolds containing 50 wt% of 45S5 Bioglass (45S5) or Sr-substituted bioactive glass (SrBG) particles by additive manufacturing technique. In addition, the scaffolds were coated with calcium phosphate (CaP). In vitro cell studies using sheep-derived bone marrow stromal cells (BMSCs) showed positive cell adhesion, growth, proliferation and up-regulation of osteogenic gene expression. The implantation of the scaffolds subcutaneously into nude rats to demonstrate the osteoinductivity potential showed the host tissue well infiltrated into the scaffolds but no mature bone formation [137]. Baheiraei et al. observed that gelatin-Sr-bioactive glass scaffolds (Gel-BG/Sr) display in vitro antibacterial properties against *Escherichia coli* and, in comparison to counterparts having no Sr ions, also against *Staphylococcus aureus*. In addition, Gel-BG/Sr scaffolds demonstrated a more enhanced deposition of newly-formed bone tissue in a rabbit calvarial bone defect in comparison to Sr-free counterpart [138]. Beside silicate BGs, borate BGs have been recently considered as attractive materials for several biomedical applications. Potential drawback may be the coordination number of boron that does not allow the formation of fully 3D structures, leading to lower resistance and higher degradation rate when in contact with body fluids. Consequently, the cytotoxicity must always be carefully evaluated [139]. In the context of borate glasses, Cui et al. reinforced poly(methylmethacrylate) (PMMA) cements and enhanced their bioactivity by incorporating Sr-containing borate BG (SrBG). The presence of SrBG promoted adhesion, migration, proliferation and collagen secretion of MC3T3-E1 cells in vitro. When the biomaterial was implanted in a tibia defect in Sprague–Dawley rats, better osseointegration at 12 weeks post-implantation was observed compared to Sr-free counterpart [140]. Fernandes et al. fabricated a composite bioactive poly-L-lactic acid (PLLA) membrane loaded with 10% (*w*/*w*) of Sr-borosilicate BG (BBG-Sr) particles (PLLA-BBG-Sr) using electrospinning. In vitro tests with bone marrow-derived mesenchymal stem cells (BM-MSCs) showed the promotion of the osteogenic differentiation with increased ALP activity and the up-regulation of osteogenic gene expression (ALP, SP7 and BGLAP) in comparison to PLLA alone [141]. The same authors highlighted the capacity of BBG-Sr particles to induce osteogenic differentiation of BM-MSCs when maintained in culture in indirect contact with the material by means of a transwell device. Indeed, favorable conditions for BM-MSC differentiation towards osteoblast-like cells and the induction of the formation of a high amount of mineralized nodules were observed [142]. Phosphate-based BGs are typically used in those clinical applications which require high dissolution rates of the implant [143]. In the case of phosphate-based glasses, Sr ions seem to locate near the chain ends preferentially [144]. Patel et al. produced discs and microspheres using Sr (0, 4, 8, 12, and 16 mol%)-substituted phosphate-based glass (PBGs). The authors reported the cytocompatibility and osteogenic potential by directly seeding MG-63 cells onto glass discs and human mesenchymal stem cells (hMSCs) onto microspheres [145].

**Table 2:** Bioactive glasses functionalized with Sr.

Material	In Vivo/In Vitro Evaluation	Results	Reference
Sr-incorporated MBGs scaffold	In vitro → bone marrow-derived stromal cells In vivo → implantation of a scaffold in critical sized femur defects in ovariectomized rats	In vitro → stimulation of proliferation and expression of osteoblast commitment markers (ALP, COL1, RUNX2, and BGLAP) In vivo → significant stimulation of new bone formation	[125]
Sr-MBG microspheres and nanoparticles	In vitro → biocomaptibility with L929 cells, inflammatory response on J774a.1 cells, and pro-osteogenic effect on Saos-2 cells	Absence of cytotoxic effect on L929 cells, absence of inflammatory response on J774a.1 cells and pro-osteogenic effect on Soas-2 cells with the stimulation of the expression of COL1, SPARC, and OPG and the downregulation of RANKL	[104]
Sr-MBGs co-grafted with hydrolysable short chain silanes containing amino (aminopropylsilanetriol) and carboxylate (carboxyethylsilanetriol) moieties	In vitro → biocompatibility with MC3T3-E1 cells and evaluation of non-specific protein adsorption	Absence of cytotoxic effect on MC3T3-E1 cells and reduction of non-specific serum protein adsorption	[105]
Sr-MBGs bio-functionalized with ICOS-Fc	In vitro → biocomaptibility with MC3T3 cells, inhibitory effect of grafted ICOS-Fc on cell migratory activity of PC-3 and U2OS cells, inhibition of osteoclast differentiation and function on monocyte-derived osteoclasts (MDOCs)	Absence of cytotoxic effects on MC3T3 cells, inhibition of PC-3 and U2OS cell migration, decrease of TRAP+ cells, and decrease of DC-STAMP, OSCAR, and NFATc1 mRNA expression	[128]
3D porous Sr-releasing, BG-based scaffold (pSrBG)	In vitro → ability of bone marrow-derived human mesenchymal stem cells to grow onto the scaffold In vivo → implantation of the scaffold in critical-sized femoral condyle defects in sheep (8 mm)	In vitro → cells attachment to scaffold inner and outer surfaces and good cell invasion and growth In vivo → promotion of the formation of mature-like well-organized lamellar neo-bone tissue	[129]
Porous nanocomposite PCL scaffolds coated with chitosan containing 15 wt% Sr-substituted BG nanoparticles (nanoparticles containing 7 wt% Sr)	In vitro → biocompatibility with MG-63 cell line	Absence of cytotoxic effects, enhanced ALP activity, and cell adhesion with healthy cell morphology	[130]
BG granules combining Sr and Mg	In vitro → biocompatibility with L929 fibroblasts and with 3D model of human BM-MSCs to predict the impact of the BG granules on bone tissue	Confirmation of material biocompatibility with L929 fibroblast cell line. Adhesion, proliferation, and osteo-lineage differentiation with 3D model of BM-MSCs	[16]
Temperature-sensitive p(N-isopropylacrylamide-co-butyl methylacrylate) nanogel with Sr containing MBGs	In vitro → preliminary evaluation with primary rat MSCs In vivo → implantation of the scaffold into femur defect in osteopenic rats	In vitro → enhanced cell proliferation and ALP activity In vivo → regeneration of critical-sized bone defects	[131]
3D bioactive composite PCL scaffolds containing 45S5 Bioglass or Sr-substituted BGs	In vitro → biocompatibility test with MC3T3 cell line	Confirmed biocompatibility and positive influence of cell attachment and proliferation. No difference in ALP activity.	[132]
Sr-containing MBG scaffold	In vitro → evaluation of stimulation of osteogenic/cementogenic differentiation of periodontal ligament cells (PDLCs)	Stimulation of ALP activity and osteogenesis/cementogenesis-related gene expression of PDLCs	[133]
3D Sr-containing MBG scaffold	In vitro → biological evaluation with MC3T3-E1 cell line	High ALP activity, enhanced expression of osteogenic markers RUNX2, OCN, BMP-2, COL1, BSP, and ECM mineralized nodules	[134]
PCL composite scaffold incorporating 10% (weight) of Sr-substituted BG particles by melt electrospinning	In vitro → biological evaluation with MC3T3-E1 cell line	Enhanced ALP activity, high expression of ALP and OCN gene, and high ECM formation	[135]
3D printed bone constructs of silk-gelatin with Sr-BG	In vitro → biological evaluation with MSCs (TVA-MSC: a specialized, immortal BMSC cell line)	Induction of osteogenic differentiation that is the up-regulation of RUNX, ALP, OPN, ON, BSP and OCN expression	[136]
PCL-based composite scaffolds containing 50 wt% of 45S5 Bioglass (45S5) or Sr-BG particles, with calcium phosphate coating	In vitro → biological evaluation with sheep-derived BMSCs In vivo → implantation of the scaffolds subcutaneously into nude rats	In vitro → positive cell adhesion, growth and proliferation and up-regulation of osteogenic gene expression In vivo → host tissue well infiltrated into the scaffolds but no mature bone formation	[137]
Gelatin-Sr-BG scaffolds (Gel-BG/Sr)	In vitro → antibacterial evaluation with *Escherichia coli* and *Staphylococcus* *aureus* In vivo → implantation in a rabbit calvarial bone defect	In vitro → antibacterial properties on *Escherichia coli* and, compared to counterparts having no Sr, also on *Staphylococcus aureus* In vivo → enhanced deposition of newly formed bone tissue in comparison to Sr-free counterpart	[138]
Poly(methylmethacrylate) cements with Sr-containing borate BG	In vitro → biological evaluation with MC3T3-E1 cell line In vivo → implantation in a tibia defect in Sprague–Dawley rats	In vitro → promotion of cell adhesion, migration, proliferation, and collagen secretion In vivo → good osseointegration after 12 weeks	[140]
Composite bioactive PLLA membrane loaded with 10% (*w/w*) of Sr-borosilicate BG particles using electrospinning	In vitro → biological evaluation with bone marrow-derived mesenchymal stem cells	Promotion of osteogenic differentiation with increased ALP activity and up-regulated osteogenic gene expression (ALP, SP7, and BGLAP) in comparison to PLLA alone	[141]
Discs and microspheres made of Sr (0, 4, 8, 12 and 16 mol%)-substituted phosphate-based glass (PBGs)	In vitro → biological evaluation of discs with MG-63 cells and microspheres with a 3D culture of human MSCs	Cell attachment and spreading confirmed for MG-63 cells with ALP activity. HMSCs attachment and colonization of the microsphere surfaces	[145]

### 9.3. Metal-Based Materials

Among the metal-based materials, the most frequently used in medicine and dentistry are titanium (Ti) and tantalum (Ta). Ti, in the form of commercially pure titanium and Ti alloys, is widely adopted in BTE applications thanks to its superior properties, specific strength, high performance, and versatility in the production of porous scaffolds, coatings, nanotubes, nanolayers, disks, and mini-screws. In addition, Ti shows excellent corrosion resistance, good hard-tissue biocompatibility, and bonding ability, alongside the ability to sustain bone formation [146]. 

Ti is chemically and mechanically stable, non-toxic and exhibits biocompatibility coupled with mechanical strength and good resistance to corrosion [147]. However, Ti does not fulfill the rapid osseointegration requirement in regenerative clinical use; consequently, surface modifications, such as the inclusion of osteogenic elements, have been applied to intensify its bone regeneration properties. The works studying metal-based materials functionalized with Sr ions are reported in Table 3.

Xin et al. developed a novel method for the controlled release of Sr from a bioactive SrTiO3 nanotube array on Ti implants, produced by a simple hydrothermal treatment of an anodized titania nanotube array. This device can release Sr at a slow rate for a long time, with good interaction with cells, i.e., cell attachment and proliferation together with the formation of HA [148]. Another example of nanotubes was developed by Mi et al.: coatings containing TiO_2_ nanotubes (NTs) incorporated with Sr on titanium (Ti) surfaces (NT-Sr) through hydrothermal treatment were tested both in vitro and in vivo. The authors observed the inhibition of osteoclast differentiation by reducing the expression of osteoclast marker genes, i.e., RANKL-induced activation of nuclear factor-κB (NF-κB), Akt, and the nuclear factor of activated T-cell cytoplasmic 1 (NFATc1) signaling pathways. NT-Sr were then implanted in ovariectomized rats, and the prevention of bone loss was observed [149]. Mumith et al. developed laser-sintered porous cylindrical Ti6Al4V implants with pore sizes of Ø 700 μm and Ø 1500 μm with electrochemically HA-coated, silicon-substituted HA (SiHA), and Sr-substituted HA (SrHA). The implants were tested in vivo in ovine femoral condylar defects for 6 weeks. The coated implants significantly promoted bone attachment to the implant surfaces with improved osseointegration compared to uncoated scaffolds [150].

Okuzu et al. evaluated the bioactivity of surface-treated Ti disks with Sr (Sr-Ti). In vitro evaluation with MC3T3-E1 cells revealed that proliferation and osteogenic differentiation, i.e., expression of integrin β1, β-catenin, and cyclin D1, osteogenic gene expression, ALP activity, and extracellular mineralization were enhanced. In vivo studies in rabbits demonstrated a major biomechanical strength and bone-implant contact for Sr-Ti compared to a Sr-free counterpart [151].

Lee et al. studied whether the surface bioactive ion modification in combination with the surface nanotopography of Ti disks exerts a positive induction of regenerative M2 macrophage polarization. The authors cultured mouse J774.A1 macrophage cells on commercially pure Ti disks with the surface functionalized with Sr ions. The regenerative M2 macrophage phenotype was observed, and the authors underlined the potential beneficial effects for the early resolution of the inflammatory state, and subsequently the favorable osteogenesis of Ti implants [152].

The surface of commercially pure Ti disks with a wet-abraded smooth or grit-blasted micro-rough surface underwent bioactive ion surface modification using Sr ions. Mesenchymal stem cells (primary murine bone marrow mesenchymal stem cells –mBMSCs- and human multipotent adipose stem cells -hASCs-) were used to evaluate the osteogenic capacity of nano-topographically and chemically modified Ti [153]. In vitro tests demonstrated that cell spreading, focal adhesion development, ALP activity, and gene expression of some integrins important for subsequent osteogenic differentiation were enhanced in mBMSCs grown on the nano/Sr surface. In addition, when hASCs were cultured on the samples, osteogenic differentiation was enhanced thanks to the presence of Sr ions [153].

Also, Zhou et al. evaluated metallic Ti disks and wires with microporous TiO_2_ coatings doped with Sr ions directly deposited on the metallic substrates using micro-arc oxidation (MAO). When biomaterials were tested with bone marrow MSCs from New Zealand rabbits, cell proliferation and osteogenesis-related gene expression were found enhanced in Sr- containing materials in comparison to Sr-free counterparts. In addition, antibacterial activity against *Staphylococcus aureus* and *Escherichia coli* was higher in the presence of Sr. When the biomaterials were implanted into femoral shafts of New Zealand male rabbits, osseointegration was observed, confirming in vitro results obtained from MSC proliferation and osteogenic differentiation tests [154].

Offermanns et al. tested in vivo, in femoral condyle defects of New Zealand White rabbits, Sr-functionalized (Ti-Sr-O) titanium implants, as an 8 mm-long cylinder with a maximum outer diameter of 3.75 mm. After an implantation period of two to twelve weeks, histological and tomographic analysis of bone-to-implant contact and bone formation were performed. The acceleration of bone apposition made the implant a potential device for endosseous implants [155]. 

Similar results in terms of enhanced osteogenic differentiation in vitro and promotion of osseointegration were collected by Wang et al. with alkali-heat treated titanium (AH-Ti) samples coated with SrTiO_3_ nanolayer by magnetron sputtering to produce a long-term Sr-releasing implant system. Different deposition durations, i.e. 30, 90, and 150 min were considered, and samples were denoted as AH-Ti/Sr30, AH-Ti/Sr90, AH-Ti/Sr150, respectively. In vitro biocompatibility with MC3T3-E1 cells demonstrated the best cytocompatibility for AH-Ti/Sr90, including cell morphology, cell viability, cell differentiation and enhanced osteogenic differentiation while hindering osteoclastogenesis. In vivo implantation in the femur of female adult Sprague Dawley rats both in normal and osteoporotic models showed that AH-Ti/Sr90 significantly promoted the osseointegration [156]. 

Encouraging results in terms of osseointegration were collected by Alenezi et al. when testing mini-screw made of cp (commercially pure) Ti grade IV deposited with SrRan loaded mesoporous titania (MT) thin coatings. Mini screws with and without SrRan were tested in tibial defects of Sprague Dawley female rats. The histological analysis revealed woven bone formation around the surface of all implants after 2 weeks, but no statistically significant differences between control and test groups were detected [157]. 

Some materials have been conceived with a combined functionalization of Sr and Ag, which grants the implant additional antibacterial properties. 

Li et al. modified the surface of a porous titanium scaffold with Sr and Ag ions (AH-Sr-AgNPs) to reduce postoperative infection and improve osteogenesis due to the release of Ag and Sr in a temporal-spatial manner. In vitro tests showed an adverse microenvironment for *Escherichia coli* and *Staphylococcus aureus* survival, M2 polarization of macrophages by using Raw 264.7 cells, and the promotion of pre-osteoblast differentiation with higher expression of ALP, RUNX2, and COL1 by using MC3T3 cells. In vivo test on infected New Zealand rabbit femoral metaphysis defects demonstrated osteogenic property of AH-Sr-AgNPs implants. In conclusion, the dual delivery of Sr and Ag has the potential of achieving an enhanced osteogenic outcome through favorable immunoregulation [158].

Another example of Sr and Ag combination is an additive manufacturing topologically ordered porous implant made from Ti-6Al-4V that is form-freedom to enable the realization of patient-specific implants. The implant was functionalized with Sr and Ag ions using plasma electrolytic oxidation: the addition of Ag to Sr completely prevented bacteria (methicillin-resistant *Staphylococcus aureus*) from adhering onto the surface of biomaterials. The long term release of ions until 28 days demonstrated the antibacterial activity behavior both in vitro and in an ex vivo murine model. Moreover, enhanced osteogenic induction in M3T3-E1 cells with higher levels of ALP activity was observed when compared with non-biofunctionalized implants [159]. Cheng et al. developed Sr and Ag loaded nanotubular structures (NT–Ag-Sr) with a controlled and prolonged release. Sr incorporation enhanced cell adhesion, migration, and proliferation of MC3T3-E1 cells cultured on the implant surface, and up-regulated the expression of osteogenic genes and induced mineralization. In parallel, the release of Ag ions allows antibacterial activity in vitro against methicillin-resistant *Staphylococcus aureus* and gram-negative bacterium *Escherichia coli* by reducing bacteria attachment. In vivo experiments after implantation of NT–Ag-Sr in tibia defects of normal and osteoporotic Sprague Dawley rats showed the accelerated formation of new bone in both osteoporosis and bone defect models, as confirmed by X-ray, micro-CT evaluation, and histomorphometric analysis [160].

**Table 3:** Metal-based materials functionalized with Sr.

Material	In Vivo/In Vitro Evaluation	Results	Reference
Bioactive SrTiO_3_ nanotube array on Ti implant	In vitro → biological evaluation with bone cells	Confirmed biocompatibility and promotion of bone cell attachment and growth	[148]
Coatings containing TiO_2_ nanotubes with Sr on titanium surfaces through hydrothermal treatment	In vitro → biological evaluation with mouse BMMCs and RAW264.7 cells In vivo → implantation in tibia defect in ovariectomized Sprague Dawley rats	In vitro → osteoclast differentiation inhibition In vivo → prevention of bone loss	[149]
Laser sintered porous cylindrical Ti6Al4V implants with 700 μm and 1500 μm pore sizes, electrochemically coated with HA, silicon-substituted HA, and Sr-substituted HA	In vivo → implantation in ovine femoral condylar defects	Coated implants significantly promoted bone attachment to the implant surface and improved osseointegration	[150]
Surface-treated Ti disks with Sr (Sr-Ti)	In vitro → biological evaluation with MC3T3-E1 cell line In vivo → implantation in tibia defect in a rabbit model	In vitro → enhanced proliferation and osteogenic differentiation with the expression of integrin β1, β-catenin, and cyclin D1, and osteogenic gene, ALP activity, extracellular mineralization In vivo → major biomechanical strength and bone-implant contact for Sr-Ti in comparison to Sr-free counterpart	[151]
Commercially pure Ti disks with surface functionalized with Sr ions	In vitro → biological evaluation with mouse J774.A1 macrophages	Induction of regenerative M2 macrophage phenotype of J774.A1 cells in nanostructured Ti surfaces	[152]
Commercially pure Ti disks with a wet-abraded smooth or grit-blasted micro rough surface functionalized with Sr ions	In vitro → biological evaluation with mesenchymal stem cells (MSCs)—primary murine BMSCs and human ASCs—	Cell spreading, focal adhesion development, ALP activity, and gene expression of integrins enhanced in mBMSCs grown on the nano Sr surface; enhanced osteogenic differentiation of hASCs in the presence of Sr	[153]
Microporous titania coatings containing Sr ions deposited onto Ti implants	In vitro → biological evaluation with bone marrow MSCs from New Zeland rabbits In vivo → implantation in femoral shafts of New Zealand male rabbits	In vitro → Sr enhanced MSCs proliferation and osteogenic differentiation In vivo → Sr enhanced implant osseointegration and new bone formation	[154]
Sr-functionalized Ti implants	In vivo → implantation in femoral condyle defect of male New Zealand White rabbits	Acceleration of bone apposition	[155]
Alkali-heat treated Ti coated with SrTiO_3_ nanolayer with different Sr content: AH-Ti/Sr30, AH-Ti/Sr90, AH-Ti/Sr150	In vitro → biological evaluation with MC3T3-E1 cell line In vivo → implantation in pile road of the femur of normal and osteoporotic female adult Sprague Dawley rats	In vitro → cytocompatibility, stimulation of osteogenic differentiation while hindering osteoclastogenesis In vivo → promotion of osseointegration both in normal and osteoporotic rat models	[156]
SrRan loaded mesoporous titania thin coatings deposited on mini-screws made of cp Ti grade IV	In vivo → implantation in bone tibia defect of Sprague Dawley female rats	Woven bone formation around the surface of all implants already after 2 weeks	[157]
Porous scaffold made of Ti with Sr and Ag ions (AH-Sr-AgNPs)	In vitro → biological evaluation with Raw 264.7 cells and MC3T3 cells and antibacterial property with *Escherichia coli* and *Staphylococcus aureus* In vivo → implantation on infected New Zealand rabbit femoral metaphysis defect	In vitro → M2 polarization of macrophages using Raw 264.7 cells and promotion of pre-osteoblast differentiation of MC3T3 cells with higher expression of ALP, RUNX2, and COL1. Promotion of an adverse microenvironment for bacterial survival In vivo → complete bone coverage and penetration into the pores of AH-Sr-AgNPs	[158]
Topologically ordered porous implant by additive manufacturing made from Ti-6Al-4V functionalized with Sr ions	In vitro → biological evaluation with MC3T3-E1 cells *Ex vivo* → antibacterial evaluation with highly virulent and multidrug-resistant *Staphylococcus aureus* by intraosseous infection model consisting of murine femora	In vitro → higher levels of ALP activity in MC3T3-E1 cells *Ex vivo* → Bactericidal effects with total eradication of both planktonic and adherent bacteria	[159]
Sr and Ag loaded nanotubular structures with controlled and prolonged release	In vitro → biological evaluation with MC3T3-E1 cells and antibacterial evaluation with methicillin-resistant *Staphylococcus aureus*, methicillin-sensitive *Staphylococcus aureus,* and *Escherichia coli* In vivo → implantation in bone defect below the epiphyseal plate of both normal and osteoporotic Sprague Dawley rats	In vitro → enhanced cell adhesion, migration, and proliferation of MC3T3-E1 cells with the up-regulated expression of osteogenic genes and induced mineralization. Antibacterial activity in vitro due to the release of Ag In vivo → accelerated formation of new bone in both osteoporotic and bone defect models	[160]

### 9.4. Polymers

Polymers used in BTE are of synthetic and natural origin. Synthetic polymers are constantly investigated in the biomedical field thanks to the possibility of tailoring their properties throughout the manufacturing process. During their synthesis, it is possible to define the purity and the reproducible chemical/mechanical properties with low costs in the production, even if on a large scale. However, the poor biocompatibility and the potential side effects due to their biodegradation products make the biological evaluation essential. Also, the effects of the long-term persistence in the body of non-resorbable polymers should be studied [161]. 

On the other hand, the natural polymers possess (i) high biocompatibility and similarity to the native ECM, (ii) bioactivity, iii) cell recognition and adhesion sites, (iv) gradual bioresorbability [162,163]. 

Natural polymers commonly used in bone-related applications include collagen, fibrin, alginate, silk and chitosan [164]. Due to the variability of the sources and to the properties depending on the extraction procedures, the manufacturing process is not always standardized. In addition, the methods used for the conversion process are often expensive and difficult to be performed. 

Pure natural polymers cannot mimic the mechanical properties of bone due to their inadequate mechanical strength and low stability, but they provide beneficial effects for cell attachment, proliferation and differentiation. Natural polymers are also more susceptible to cross-contamination and immunogenicity. 

To overcome these potential drawbacks, synthetic polymers with more tunable characteristics, such as polylactic acid (PLA), poly(glycolic acid) PGA, poly lactic-co-glycolic acid (PLGA), and polycaprolactone (PCL), are largely employed [99]. 

The possibility to combine different polymers and to functionalize with inorganic phases at multiple levels to tailor hybrid materials for bone regenerative applications is an interesting strategy. In addition, the physicochemical properties and the bioactivity can be controlled, as well as the final form of the material, i.e. 3D scaffolds, hydrogels, microspheres, and their composites [165]. The works studying polymer functionalized with Sr are reported in Table 4.

Collagen scaffolds have been reinforced with Sr−graphene oxide to improve mechanical properties, allowing a long-term release of Sr and consequent enhancement of bone regeneration in a critical-size defect in rats after 12 weeks of implantation [166]. 

Montalbano et al. fabricated MBGs containing 4% molar of Sr (Sr/Ca/Si = 4/11/85) exploiting a base-catalyzed sol-gel method. Sr-doped MBGs were mixed with a 1.5 wt % collagen solution to create a collagen-based hybrid material for further exploitation with 3D-printing technology. In a first work, the hybrid formulation was cross-linked with 4-star poly (ethylene glycol) ether tetrasuccinimidyl glutarate (4-StarPEG) and tested in vitro with MG-63 cells. The seeding of MG-63 on the top of bulk material demonstrated cell adhesion with good viability [167]. In a second work, the hybrid formulation was cross-linked with genipin dissolved in 70% ethanol. The biocompatibility of the hybrid system was confirmed by using MG-63 and Saos-2 cell lines. In addition, the use of genipin in 70% EtOH as crosslinking solution resulted in a significant decrease of Sr release during the crosslinking reaction time [168]. Successively, the same authors prepared 1.5% wt collagen hydrogel containing MBGs incorporating 4% molar of Sr (MBG_Sr4%) produced via the sol-gel route. The bulk samples were tested by employing an indirect co-culture of human osteoblasts and osteoclast precursors. Sr-enriched mesoporous BGs have structural and physicochemical properties that support the viability and proliferation of co-cultured human bone-derived cells, with multiple signals differently affecting the osteoblast and the osteoclast precursors [169]. 

Fenbo et al. developed a chondroitin sulfate/silk fibroin blended membrane with a microporous structure loaded with different concentrations of Sr. In vitro results demonstrated the downregulation of pro-inflammatory cytokines in RAW 264.7 cells and the upregulation of osteogenic factors in human osteoblasts [170].

Similarly, Lu et al. evaluated in vitro and in vivo the feasibility of Sr-loaded silk fibroin nanofibrous membrane (Sr-SFM) for guided bone regeneration. The authors observed in vitro the enhancement of cell numbers and ALP activity of rat bone marrow stromal cells (rBMSCs) cultured on Sr-SFM compared to Sr-free counterpart and a more pronounced bone formation when implanted in rat calvarial defect model after 6 weeks of healing [171]. Luz et al. developed a hybrid material composed of bacterial cellulose (BC) and HA loaded with Sr. The authors observed that the delivery of Sr can be modulated during bone repair depending on the strategy of Sr functionalization into the matrix of the material [172]. Cheng et al. developed a Sr-containing scaffold (CPB/PCL/Sr) based on superficially porous calcined porcine bone (CPB) by a sequential coating of SrCl2 and polycaprolactone (PCL), with improved bone-forming ability as a promising alternative to bone defect repair materials. When tested with human MSCs, CPB/PCL/Sr scaffold induced a remarkable osteogenic differentiation of MSCs, while when implanted in a bone defect in tibia defect of male SD rats, a higher bone mass formation was observed in comparison to Sr-free counterpart [173].

Concerning PCL, Lino et al. developed a blend of PCL and poly(diisopropyl fumarate) enriched with 1% or 5% Sr, to be tested both in vitro and in vivo to find that the low Sr-containing blend induces an improved bone tissue regeneration. Indeed, in vitro blend with 5% Sr was pro-inflammatory and anti-osteogenic, while blend with 1% Sr was not cytotoxic on cultured macrophages and demonstrated an improved osteocompatibility with primary cultures of bone marrow stromal cells. In vivo experiments showed a significantly increased bone tissue regeneration and improved fibrous bridging for the blend with 1% Sr [174]. 

Again with PCL polymer, Prabha et al. developed a PCL–laponite–SrRan composite scaffold (PLS3) and observed cell growth and osteogenic differentiation in vitro when tested with human telomerase immortalized bone marrow derived skeletal stem cell line, and vascularized ectopic bone formation when hMSC loaded-PLS3 was implanted subcutaneously in NOD.CB17-Prkdcscid/J mice [175].

In the last years, microparticles have been investigated as a carrier for cartilage and bone tissue regenerative approaches [176]. In this scenario, Sr can be loaded into microparticles to control the spatial and temporal release through the biodegradation of the microparticles. In addition, the functionalization of the material surface with Sr-doped microparticles may be performed to integrate multiple functions into one design. 

Wei et al. developed microparticles made of a copolymer consisting of PLLA and poly(ethyl glycol) (PEG) blocks containing both vancomycin and Sr-doped apatite to provide antibacterial effect and osteo-promoting activity. Strong antibacterial effect against *Staphylococcus aureus* and excellent cell compatibility with bone marrow mesenchymal stromal cells (BMSCs) derived from Sprague-Dawley rat were demonstrated. In addition, Sr enhanced the angiogenic and osteogenic expressions of MSCs, while the subcutaneous injection of the microspheres into the rabbit’s back induced neovascularization and ectopic osteogenesis. Moreover, the implantation of the microparticles in an infected rabbit femoral condyle defect (created with *Staphylococcus aureus* infection) resulted in significant antibacterial activity in vivo and achievement of an efficient new bone deposition [177]. 

Membrane scaffold composed of a matrix of ionically cross-linked chitosan and microparticles of PCL containing 5 wt% Sr salts demonstrated good biocompatible properties. When tested in vitro with MG-63 cells and hBMSCs, the absence of cytotoxicity, good cell adhesion and spreading, and higher ALP activity were recorded. When implanted in a subcutaneous model in rats, Sr-containing membrane showed a biocompatible behavior inducing less fibrosis with a thinner fibrous tissue [178].

Wang et al. fabricated a near-infrared (NIR) light-triggered drug delivery system incorporating black phosphorus (BPs) and SrCl2 with the PLGA microspheres (BP-SrCl2/PLGA microspheres). In vitro evaluation demonstrated excellent cell viability and biodegradability and a good bone regeneration capability after implantation in femoral defects of Wistar rats. Good vascularization, cell integration, and migration into deeper scaffold layers were also observed [179].

Also, the strategy of encapsulating Sr in a polymer has been exploited. Sr has been encapsulated in PLA microcapsules and maintained in an osteogenic medium for more than 121 days. The precipitation of biomimetic CaP on the surface and in the pores of microcapsules was obtained as proof of the potential of Sr to promote bone deposition [180]. In addition, the evaluation of cell viability using MG-63 cells showed no evidence of the cytotoxic effect of the microcapsule extracts [180].

**Table 4:** Polymers functionalized with Sr.

Material	In Vivo/In Vitro Evaluation	Results	Reference
Collagen scaffold reinforced with Sr−graphene oxide	In vitro → biological evaluation with human adipose-derived stem cells and human umbilical vein endothelial cell In vivo → implantation in a critical-size bone defect in rat	In vitro → cell adhesion and spreading, marked mineralization and enhanced ALP activity, with enhanced expression of VEGF and BMP-2, tube formation and angiogenesis In vivo → enhancement of bone regeneration after 12 weeks of implantation	[166]
Collagen-based material with Sr-doped MBGs	In vitro → biological evaluation with MG-63 cells	High biocompatibility	[167]
Collagen-based material with Sr-doped MBGs	In vitro → biological evaluation with MG-63 and Saos-2 cells	High biocompatibility	[168]
Collagen-based material with Sr-doped MBGs	In vitro → biological evaluation with an indirect co-culture of human osteoblasts and osteoclast precursors	High biocompatibility and ability to support viability and proliferation of human bone-derived cells	[169]
Chondroitin sulfate/silk fibroin blended membrane with microporous structure loaded with different concentrations of Sr	In vitro → biological evaluation with RAW 264.7 cells and human osteoblasts	Downregulation of pro-inflammatory cytokines in RAW 264.7 cells and upregulation of osteogenic factors in human osteoblasts	[170]
Sr-loaded silk fibroin nanofibrous membrane (Sr-SFM) (1%, 5%, and 10% Sr)	In vitro → biological evaluation with rat bone marrow stromal cells In vivo → implantation in rat calvarial defect model	In vitro → enhancement in cell numbers, cell adhesion and ALP activity in Sr-SFM in comparison to Sr-free counterpart In vivo → pronounced bone formation after 6 weeks (especially in 10% Sr-SFM group)	[171]
Porous calcined porcine bone scaffold coated with SrCl2 and PCL	In vitro → biological evaluation with human fetal mesenchymal stem cells (MSCs) In vivo → implantation in a bone defect in the tibia of male SD rats	In vitro → osteogenic differentiation of MSCs In vivo → a better new bone formation in the presence of Sr	[173]
Blend of PCL and poly(diisopropyl fumarate) enriched with 1% or 5% Sr	In vitro → biological evaluation with bone marrow stromal cells from young male WKAH/Hok Wistar rats In vivo → implantation in a circular bone defect in parietal bones of WKAH/Hok Wistar rats	In vitro → better proliferation and COL1 and ALP expression for blend + 1% Sr in comparison to Blend + 5% Sr In vivo → increased bone tissue regeneration and improved fibrous bridging for blend + 1% Sr	[174]
PCL–laponite–SrRan composite scaffold	In vitro → biological evaluation with human telomerase immortalized bone marrow derived skeletal stem cell line (hMSC-TERT) In vivo → implantation of hMSC-seeded PLS3 subcutaneously in SCID mice	In vitro → cell growth and osteogenic differentiation In vivo → vascularized ectopic bone formation	[175]
Microparticles composed of PLLA and PEG copolymer containing vancomycin and strontium-doped apatite	In vitro → biological evaluation with bone marrow mesenchymal stromal cells (BMSCs) derived from Sprague-Dawley rat In vivo → subcutaneous implantation in pockets in rabbit backs (ectopic site); implantation in a cylindrical infected bone defect in rabbit’s lateral femoral condyle	In vitro → antibacterial effect against *Staphylococcus aureus* and excellent biocompatibility with BMSCs In vivo → induction of neovascularization and ectopic osteogenesis; significant antibacterial activity and efficient new bone deposition	[177]
Membrane scaffold composed of a matrix of ionically cross-linked chitosan and microparticles of PCL containing 5 wt% Sr salts	In vitro → biological evaluation with MG-63 cells and hBMSCs In vivo → implantation in a subcutaneous model in rats	In vitro → absence of cytotoxicity, better adhesion and spreading, and higher ALP activity with MG-63 cells; good adhesion and proliferation together with higher ALP level for hBMSCs In vivo → biocompatible behaviour especially for Sr-containing membrane: less development of fibrosis with a thinner fibrous tissue	[178]
Black phosphorus (BPs) and SrCl2 with PLGA microspheres (BP-SrCl2/PLGA microspheres) as a near-infrared light-triggered drug delivery system	In vitro → biological evaluation with hMSCs In vivo → implantation in femoral defects of Wistar rats	In vitro → excellent cell viability, osteoblastic differentiation, and biodegradability In vivo → good bone regeneration capability	[179]
Sr encapsulated in PLA microcapsules	In vitro → biological evaluation with MG-63 cells	Absence of cytotoxic effect of microcapsule extracts	[180]

## 10. Discussion

Currently, the functionalization of materials with biologically active ions, including Sr, is an emerging technology: inorganic trace elements are added to biomaterials to improve their biological and physico-mechanical performance, and to promote skeletal tissue regeneration [181].

The introduction of Sr ions in biomaterials has attracted interest in the last years: the similarity of atomic and ionic properties with Ca is the strength of Sr. 

Sr ions interact with calcium-sensing receptors of bone cells, and by acting on the molecular pathways of osteoblasts and osteoclasts, as well as on osteoblast precursors, positively stimulate bone formation while negatively influencing bone resorption, as proven by in vitro and in vivo studies [129]. In addition, Sr becomes a semi-physiological component of the bone tissue by bonding the mineral crystals of bone [182]. 

In the present review, biomaterials for bone regeneration incorporating Sr have been reviewed according to the types of materials used in BTE, i.e., calcium phosphates, bioactive glasses, metal-based materials, and polymers [183].

The main criterion for the article selection of this review has been the presence of a consistent biological evaluation of the in vitro/in vivo performance of such materials. 

Thus, the works were organized into the four above-mentioned groups, and the main results were summarized in the Tables in order to easily display the material characterization, the in vitro/in vivo evaluation, and the main biological results achieved. 

Sr-doped calcium phosphate ceramics are often evaluated in vivo in animal defect models with encouraging results on active bone formation and successful healing of bone defects. When tested in vitro, Sr-doped CaP are able to support the adhesion and proliferation of osteogenic-lineage cells and to promote osteogenic differentiation. 

Sr-doped bioactive glasses may be used alone as microspheres and nanoparticles, and in combination with polymers, such as PCL or PLLA, to produce composite materials. As final materials, 3D-scaffolds may be obtained, by 3D printing or electrospinning. BGs are generally tested in vitro to evaluate biocompatibility, cytotoxic effects, promotion of cell proliferation and osteogenic differentiation. 

Metal-based materials with Sr functionalization are often made of titanium. This type of material is available in different forms, i.e. cylinder, screw, porous scaffold, nanotubes, etc. Alongside the in vitro evaluation to verify biocompatibility and promotion of cell adhesion, in vivo evaluation is generally performed, due to the extended lifetime of the implant within the body.

Polymers functionalized with Sr reported in this review are both natural and synthetic. Collagen is the most studied natural polymer, usually reinforced with other materials such as bioactive glasses, while among the synthetic polymers PCL, PLGA, PLA are frequently adopted. 

As a general rule, promising in vitro data are obtained for all the four types of biomaterials, with the absence of cytotoxic effects, i.e. biocompatibility, tested as initial crucial aspects of regenerative therapies. Thanks to the presence and release of Sr ions, osteogenic differentiation of bone cell precursors is stimulated, with the increase of osteogenic marker expression, such as ALP, RUNX2, COL1, OPN, OCN in comparison to Sr-free counterparts [184]. Sr also negatively affects osteoclastogenesis and bone resorption. When tested in vivo in bone defect models, most of the materials result in new bone formation and osseointegration of the material, as well as tissue mineralization, in comparison to Sr-free counterparts. 

Limits of the in vitro assays include the inability to reproduce the spatial and temporal release of Sr within tissues and the large use of cell lines, which resemble primary bone cells but do not behave exactly as primary human cells. Also, within the in vitro/in vivo studies, Sr doses should resemble the local amount released in bone following Sr administration, but physiological values are hardly known and experimentally reproduced. 

However, even if the papers analyzed in this review report several benefits, further studies and assays are required to disclose the local effects of the Sr-release to take more advantage of its use. 

A better understanding of the activity and the dose-dependent effects will allow the translation of the in vitro results of synthetic biomaterials into the clinical setting. Precise mechanisms concerning Sr-induced osteogenic outcomes must be explored to design clinically feasible Sr-based biomaterials.

The strategy to add trace elements to implantable biomaterials confirms a worthwhile strategy for the direct delivery to the defect site. Positive stimuli for bone healing throughout the lifetime of the implant are provided [5].

## 11. Conclusions

Promising results have been reported in the last two decades upon strontium-enriched biomaterials: a better performance both in vivo and in vitro has been observed in the presence of strontium compared to the strontium-free counterpart. 

The functionalization of biomaterials with strontium for bone regenerative strategies has been shown to improve bone healing by enhancing local bone regeneration.

Thus, new strategies to incorporate strontium into biomaterials and control the local release are necessary points to be addressed, to improve the actual delivery systems and overcome the limitations.

The works reported in the review, together with new insights into the use of strontium-enriched biomaterials, demonstrate the potential of advanced tissue engineering therapies in bone fractures regeneration. 

Strontium-enriched biomaterials are confirmed to be a good strategy to administrate strontium ions avoiding the systemic critical issues of strontium ranelate. 

## References

[1] Murray T. (1993). Elementary Scots the Discovery of Strontium. Scott. Med. J..

[2] Watts P., Howe P. (2010). Strontium and Strontium Compounds.

[3] Nielsen S.P. (2004). The biological role of strontium. Bone.

[4] Andersen O.Z., Offermanns V., Sillassen M., Almtoft K.P., Andersen I.H., Sørensen S., Jeppesen C.S., Kraft D.C., Bøttiger J., Rasse M. (2013). Accelerated bone ingrowth by local delivery of strontium from surface functionalized titanium implants. Biomaterials.

[5] Bose S., Fielding G., Tarafder S., Bandyopadhyay A. (2013). Trace element doping in calcium phosphate ceramics to Understand osteogenesis and angiogenesis. Trends Biotechnol..

[6] Reginster J.-Y., Lecart M.-P., Deroisy R., Lousberg C. (2004). Strontium ranelate: A new paradigm in the treatment of osteoporosis. Expert Opin. Investig. Drugs.

[7] Reginster J.Y., Seeman E., De Vernejoul M.C., Adami S., Compston J., Phenekos C., Devogelaer J.P., Curiel M.D., Sawicki A., Goemaere S. (2005). Strontium Ranelate Reduces the Risk of Nonvertebral Fractures in Postmenopausal Women with Osteoporosis: Treatment of Peripheral Osteoporosis (TROPOS) Study. J. Clin. Endocrinol. Metab..

[8] Marie P.J. (2005). Strontium as therapy for osteoporosis. Curr. Opin. Pharmacol..

[9] Marie P.J., Hott M., Modrowski D., De Pollak C., Guillemain J., Deloffre P., Tsouderos Y. (1993). An uncoupling agent containing strontium prevents bone loss by depressing bone resorption and maintaining bone formation in estrogen-deficient rats. J. Bone Miner. Res..

[10] Meunier P.J., Roux C., Seeman E., Ortolani S., Badurski J.E., Spector T.D., Cannata-Andía J.B., Balogh A., Lemmel E.-M., Pors-Nielsen S. (2004). The Effects of Strontium Ranelate on the Risk of Vertebral Fracture in Women with Postmenopausal Osteoporosis. N. Engl. J. Med..

[11] Shorr E., Carter A.C. (1952). The usefulness of strontium as an adjuvant to calcium in the remineralization of the skeleton in man. Bull. Hosp. Jt. Dis..

[12] Morohashi T., Izumisawa T., Matsumoto A., Yamada S. (1993). The effects of stable strontium on calcium metabolism: I. Kinetic analysis of calcium metabolism in strontium-fed rats. J. Bone Miner. Metab..

[13] Reginster J.Y., Deroisy R., Dougados M., Jupsin I., Colette J., Roux C. (2002). Prevention of Early Postmenopausal Bone Loss by Strontium Ranelate: The Randomized, Two-Year, Double-Masked, Dose-Ranging, Placebo-Controlled PREVOS Trial. Osteoporos. Int..

[14] Pilmane M., Salma-Ancane K., Loca D., Locs J., Berzina-Cimdina L. (2017). Strontium and strontium ranelate: Historical review of some of their functions. Mater. Sci. Eng. C.

[15] Kołodziejska B., Stępień N., Kolmas J. (2021). The Influence of Strontium on Bone Tissue Metabolism and Its Application in Osteoporosis Treatment. Int. J. Mol. Sci..

[16] Bellucci D., Veronesi E., Strusi V., Petrachi T., Murgia A., Mastrolia I., Dominici M., Cannillo V. (2019). Human Mesenchymal Stem Cell Combined with a New Strontium-Enriched Bioactive Glass: An ex-vivo Model for Bone Regeneration. Materials.

[17] Kruppke B., Ray S., Alt V., Rohnke M., Kern C., Kampschulte M., Heinemann C., Budak M., Adam J., Döhner N. (2020). Gelatin-Modified Calcium/Strontium Hydrogen Phosphates Stimulate Bone Regeneration in Osteoblast/Osteoclast Co-Culture and in Osteoporotic Rat Femur Defects—In Vitro to In Vivo Translation. Molecules.

[18] Xue W., Moore J.L., Hosick H.L., Bose S., Bandyopadhyay A., Lu W.W., Cheung K.M.C., Luk K.D.K. (2006). Osteoprecursor cell response to strontium-containing hydroxyapatite ceramics. J. Biomed. Mater. Res. Part A.

[19] Lourenço A.H., Neves N., Machado C., Sousa S.R., Lamghari M., Barrias C., Cabral A.T., Barbosa M.A., Ribeiro C.C. (2017). Injectable hybrid system for strontium local delivery promotes bone regeneration in a rat critical-sized defect model. Sci. Rep..

[20] Marx D., Yazdi A.R., Papini M., Towler M. (2020). A review of the latest insights into the mechanism of action of strontium in bone. Bone Rep..

[21] Blake G.M., Fogelman I. (2006). Strontium ranelate: A novel treatment for postmenopausal osteoporosis: A review of safety and efficacy. Clin. Interv. Aging.

[22] Stepan J.J. (2013). Strontium ranelate: In search for the mechanism of action. J. Bone Miner. Metab..

[23] Blake G.M., Compston J.E., Fogelman I. (2009). Could strontium ranelate have a synergistic role in the treatment of osteoporosis?. J. Bone Miner. Res..

[24] Donneau A.-F., Reginster J.-Y. (2013). Cardiovascular safety of strontium ranelate: Real-life assessment in clinical practice. Osteoporos. Int..

[25] (2014). European Medicines Agency Protelos/Osseor to Remain Available but with Further Restrictions. https://www.ema.europa.eu/en/news/recommendation-restrict-use-protelos-osseor-strontium-ranelate.

[26] European Medicines Agency (2014) Strontium Ranelate (2014). Summary of Product Characteristics. https://www.ema.europa.eu/en/medicines/human/EPAR/protelos.

[27] Compston J. (2014). Strontium ranelate lives to fight another day. Maturitas.

[28] Audran M., Jakob F.J., Palacios S., Brandi M.-L., Bröll H., Hamdy N.A.T., Mccloskey E.V. (2013). A large prospective European cohort study of patients treated with strontium ranelate and followed up over 3 years. Rheumatol. Int..

[29] Reginster J.-Y., Brandi M.-L., Cannata-Andía J.B., Cooper C., Cortet B., Feron J.-M., Genant H., Palacios S., Ringe J.D., Rizzoli R. (2015). The position of strontium ranelate in today’s management of osteoporosis. Osteoporos. Int..

[30] Pin J.-P., Galvez T., Prézeau L. (2003). Evolution, structure, and activation mechanism of family 3/C G-protein-coupled receptors. Pharmacol. Ther..

[31] Brown E.M., MacLeod R.J. (2001). Extracellular Calcium Sensing and Extracellular Calcium Signaling. Physiol. Rev..

[32] Hannan F.M., Kallay E., Chang W., Brandi M.L., Thakker R.V. (2018). The calcium-sensing receptor in physiology and in calcitropic and noncalcitropic diseases. Nat. Rev. Endocrinol..

[33] Geng Y., Mosyak L., Kurinov I., Zuo H., Sturchler E., Cheng T.C., Subramanyam P., Brown A.P., Brennan S.C., Mun H.-C. (2016). Author response: Structural mechanism of ligand activation in human calcium-sensing receptor. Elife.

[34] Chattopadhyay N., Quinn S.J., Kifor O., Ye C., Brown E.M. (2007). The calcium-sensing receptor (CaR) is involved in strontium ranelate-induced osteoblast proliferation. Biochem. Pharmacol..

[35] Brennan S.C., Thiem U., Roth S., Aggarwal A., Fetahu I.S., Tennakoon S., Gomes A.R., Brandi M.L., Bruggeman F., Mentaverri R. (2013). Calcium sensing receptor signalling in physiology and cancer. Biochim. Biophys. Acta—Mol. Cell Res..

[36] Goltzman D., Hendy G.N. (2015). The calcium-sensing receptor in bone—Mechanistic and therapeutic insights. Nat. Rev. Endocrinol..

[37] Brown E.M. (2003). Is the calcium receptor a molecular target for the actions of strontium on bone?. Osteoporos. Int..

[38] Fromigué O., Haã¿ E., Barbara A., Petrel C., Traiffort E., Ruat M., Marie P.J. (2009). Calcium sensing receptor-dependent and receptor-independent activation of osteoblast replication and survival by strontium ranelate. J. Cell. Mol. Med..

[39] Pi M., Quarles L.D. (2004). A Novel Cation-Sensing Mechanism in Osteoblasts Is a Molecular Target for Strontium. J. Bone Miner. Res..

[40] Marie P.J., Ammann P., Boivin G., Rey C. (2001). Mechanisms of Action and Therapeutic Potential of Strontium in Bone. Calcif. Tissue Res..

[41] Dahl S.G., Allain P., Marie P., Mauras Y., Boivin G., Ammann P., Tsouderos Y., Delmas P., Christiansen C. (2001). Incorporation and distribution of strontium in bone. Bone.

[42] O’Flaherty E.J. (1992). Modeling bone mineral metabolism, with special reference to calcium and lead. Neurotoxicology.

[43] Johnson A.R., Armstrong W.D., Singer L. (1968). The incorporation and removal of large amounts of strontium by physiologic mechanisms in mineralized tissues of the rat. Calcif. Tissue Res..

[44] Boivin G., Deloffre P., Perrat B., Panczer G., Boudeulle M., Mauras Y., Allain P., Tsouderos Y., Meunier P.J. (2009). Strontium distribution and interactions with bone mineral in monkey iliac bone after strontium salt (S 12911) administration. J. Bone Miner. Res..

[45] Doublier A., Farlay D., Khebbab M.T., Jaurand X., Meunier P.J., Boivin G. (2011). Distribution of strontium and mineralization in iliac bone biopsies from osteoporotic women treated long-term with strontium ranelate. Eur. J. Endocrinol..

[46] Nelson D.G.A., Featherstone J.D.B., Duncan J.F., Cutress T.W. (1982). Paracrystalline Disorder of Biological and Synthetic Carbonate-substituted Apatites. J. Dent. Res..

[47] LeGeros R.Z. (1990). Chemical and Crystallographic Events in the Caries Process. J. Dent. Res..

[48] Berebichez-Fridman R., Gómez-García R., Granados-Montiel J., Berebichez-Fastlicht E., Olivos-Meza A., Granados J., Velasquillo C., Ibarra C. (2017). The Holy Grail of Orthopedic Surgery: Mesenchymal Stem Cells—Their Current Uses and Potential Applications. Stem Cells Int..

[49] Dominici M., Le Blanc K., Mueller I., Slaper-Cortenbach I., Marini F.C., Krause D.S., Deans R.J., Keating A., Prockop D.J., Horwitz E.M. (2006). Minimal criteria for defining multipotent mesenchymal stromal cells. The International Society for Cellular Therapy position statement. Cytotherapy.

[50] Oryan A., Kamali A., Moshiri A., Eslaminejad M.B. (2017). Role of Mesenchymal Stem Cells in Bone Regenerative Medicine: What Is the Evidence?. Cells Tissues Organs.

[51] Yang F., Yang D., Tu J., Zheng Q., Cai L., Wang L. (2011). Strontium Enhances Osteogenic Differentiation of Mesenchymal Stem Cells and In Vivo Bone Formation by Activating Wnt/Catenin Signaling. Stem Cells.

[52] Su T.-R., Huang T.-H., Kao C.-T., Ng H.Y., Chiu Y.-C., Hsu T.-T. (2020). The Calcium Channel Affect Osteogenic Differentiation of Mesenchymal Stem Cells on Strontium-Substituted Calcium Silicate/Poly-ε-Caprolactone Scaffold. Processes.

[53] Li Y., Li J., Zhu S., Luo E., Feng G., Chen Q., Hu J. (2012). Effects of strontium on proliferation and differentiation of rat bone marrow mesenchymal stem cells. Biochem. Biophys. Res. Commun..

[54] Sila-Asna M., Bunyaratvej A., Maeda S., Kitaguchi H., Bunyaratavej N. (2007). Osteoblast differentiation and bone formation gene expression in strontium-inducing bone marrow mesenchymal stem cell. Kobe J. Med. Sci..

[55] Canalis E. (1996). The divalent strontium salt S12911 enhances bone cell replication and bone formation in vitro. Bone.

[56] Peng S., Liu X.S., Huang S., Li Z., Pan H., Zhen W., Luk K.D.K., Guo X.E., Lu W.W. (2011). The cross-talk between osteoclasts and osteoblasts in response to strontium treatment: Involvement of osteoprotegerin. Bone.

[57] Zhu L.-L., Zaidi S., Peng Y., Zhou H., Moonga B.S., Blesius A., Dupin-Roger I., Zaidi M., Sun L. (2007). Induction of a program gene expression during osteoblast differentiation with strontium ranelate. Biochem. Biophys. Res. Commun..

[58] Choudhary S., Halbout P., Alander C., Raisz L., Pilbeam C. (2007). Strontium Ranelate Promotes Osteoblastic Differentiation and Mineralization of Murine Bone Marrow Stromal Cells: Involvement of Prostaglandins. J. Bone Miner. Res..

[59] Brennan T.C., Rybchyn M.S., Green W., Atwa S., Conigrave A.D., Mason R.S. (2009). Osteoblasts play key roles in the mechanisms of action of strontium ranelate. J. Cereb. Blood Flow Metab..

[60] Choudhary S., Wadhwa S., Raisz L.G., Alander C., Pilbeam C.C. (2003). Extracellular Calcium Is a Potent Inducer of Cyclo-oxygenase-2 in Murine Osteoblasts Through an ERK Signaling Pathway. J. Bone Miner. Res..

[61] Barbara A., Delannoy P., Denis B.G., Marie P.J. (2004). Normal matrix mineralization induced by strontium ranelate in MC3T3-E1 osteogenic cells. Metabolism.

[62] Fonseca J.E. (2008). Rebalancing bone turnover in favour of formation with strontium ranelate: Implications for bone strength. Rheumatology.

[63] Bonnelye E., Chabadel A., Saltel F., Jurdic P. (2008). Dual effect of strontium ranelate: Stimulation of osteoblast differentiation and inhibition of osteoclast formation and resorption in vitro. Bone.

[64] Wornham D.P., Hajjawi M.O., Orriss I.R., Arnett T.R. (2014). Strontium potently inhibits mineralisation in bone-forming primary rat osteoblast cultures and reduces numbers of osteoclasts in mouse marrow cultures. Osteoporos. Int..

[65] Verberckmoes S.C., De Broe M.E., D’Haese P.C. (2003). Dose-dependent effects of strontium on osteoblast function and mineralization. Kidney Int..

[66] Rybchyn M.S., Slater M., Conigrave A.D., Mason R.S. (2011). An Akt-dependent Increase in Canonical Wnt Signaling and a Decrease in Sclerostin Protein Levels Are Involved in Strontium Ranelate-induced Osteogenic Effects in Human Osteoblasts. J. Biol. Chem..

[67] Saidak Z., Marie P.J. (2012). Strontium signaling: Molecular mechanisms and therapeutic implications in osteoporosis. Pharmacol. Ther..

[68] Marie P.J., Miraoui H., Sévère N. (2012). FGF/FGFR signaling in bone formation: Progress and perspectives. Growth Factors.

[69] Caverzasio J. (2008). Strontium ranelate promotes osteoblastic cell replication through at least two different mechanisms. Bone.

[70] Caverzasio J., Thouverey C. (2011). Activation of FGF Receptors is a new Mechanism by which Strontium Ranelate Induces Osteoblastic Cell Growth. Cell. Physiol. Biochem..

[71] Pi M., Faber P., Ekema G., Jackson P.D., Ting A., Wang N., Fontilla-Poole M., Mays R.W., Brunden K.R., Harrington J.J. (2005). Identification of a Novel Extracellular Cation-sensing G-protein-coupled Receptor. J. Biol. Chem..

[72] Pi M., Chen L., Huang M.-Z., Zhu W., Ringhofer B., Luo J., Christenson L., Li B., Zhang J., Jackson P.D. (2008). GPRC6A Null Mice Exhibit Osteopenia, Feminization and Metabolic Syndrome. PLoS ONE.

[73] Pi M., Zhang L., Lei S.-F., Huang M.-Z., Zhu W., Zhang J., Shen H., Deng H.-W., Quarles L.D. (2009). Impaired Osteoblast Function inGPRC6ANull Mice. J. Bone Miner. Res..

[74] Tan S., Zhang B., Zhu X., Ao P., Guo H., Yi W., Zhou G.-Q. (2014). Deregulation of Bone Forming Cells in Bone Diseases and Anabolic Effects of Strontium-Containing Agents and Biomaterials. BioMed Res. Int..

[75] Baron R., Tsouderos Y. (2002). In vitro effects of S12911-2 on osteoclast function and bone marrow macrophage differentiation. Eur. J. Pharmacol..

[76] Descartes M., Sillence D.O. (2013). Disorders of Bone Density, Volume, and Mineralization. Emery and Rimoin’s Principles and Practice of Medical Genetics.

[77] Billecocq A., Emanuel J.R., Levenson R., Baron R. (1990). 1 alpha,25-dihydroxyvitamin D3 regulates the expression of carbonic anhydrase II in nonerythroid avian bone marrow cells. Proc. Natl. Acad. Sci. USA.

[78] Lomri A., Baron R. (1992). 1 alpha,25-dihydroxyvitamin D3 regulates the transcription of carbonic anhydrase II mRNA in avian myelomonocytes. Proc. Natl. Acad. Sci. USA.

[79] Lakkakorpi P.T., Väänänen H.K. (1996). Cytoskeletal changes in osteoclasts during the resorption cycle. Microsc. Res. Tech..

[80] Schumacher M., Wagner A.S., Kokesch-Himmelreich J., Bernhardt A., Rohnke M., Wenisch S., Gelinsky M. (2016). Strontium substitution in apatitic CaP cements effectively attenuates osteoclastic resorption but does not inhibit osteoclastogenesis. Acta Biomater..

[81] Takahashi N., Sasaki T., Tsouderos Y., Suda T. (2003). S 12911-2 Inhibits Osteoclastic Bone Resorption In Vitro. J. Bone Miner. Res..

[82] Caudrillier A., Hurtel-Lemaire A.-S., Wattel A., Cournarie F., Godin C., Petit L., Petit J.-P., Terwilliger E., Kamel S., Brown E.M. (2010). Strontium Ranelate Decreases Receptor Activator of Nuclear Factor-κB Ligand-Induced Osteoclastic Differentiation In Vitro: Involvement of the Calcium-Sensing Receptor. Mol. Pharmacol..

[83] Coulombe J., Faure H., Robin B., Ruat M. (2004). In vitro effects of strontium ranelate on the extracellular calcium-sensing receptor. Biochem. Biophys. Res. Commun..

[84] Hurtel-Lemaire A.S., Mentaverri R., Caudrillier A., Cournarie F., Wattel A., Kamel S., Terwilliger E.F., Brown E.M., Brazier M. (2009). The Calcium-sensing Receptor Is Involved in Strontium Ranelate-induced Osteoclast Apoptosis. J. Biol. Chem..

[85] Boyce B.F., Xing L. (2007). The RANKL/RANK/OPG pathway. Curr. Osteoporos. Rep..

[86] Atkins G.J., Welldon K.J., Halbout P., Findlay D.M. (2008). Strontium ranelate treatment of human primary osteoblasts promotes an osteocyte-like phenotype while eliciting an osteoprotegerin response. Osteoporos. Int..

[87] Zhang W., Shen Y., Pan H., Lin K., Liu X., Darvell B.W., Lu W.W., Chang J., Deng L., Wang D. (2011). Effects of strontium in modified biomaterials. Acta Biomater..

[88] Forsgren J., Engqvist H. (2010). A novel method for local administration of strontium from implant surfaces. J. Mater. Sci. Mater. Med..

[89] Pina S., Torres P.M.C., Goetz-Neunhoeffer F., Neubauer J., Ferreira J.M.F. (2010). Newly developed Sr-substituted α-TCP bone cements. Acta Biomater..

[90] Glenske K., Donkiewicz P., Köwitsch A., Milosevic-Oljaca N., Rider P., Rofall S., Franke J., Jung O., Smeets R., Schnettler R. (2018). Applications of Metals for Bone Regeneration. Int. J. Mol. Sci..

[91] Jiwoon J., Kim J.H., Shim J.H., Hwang N.S., Heo C.Y. (2019). Bioactive calcium phosphate materials and applications in bone regeneration. Biomater. Res..

[92] Liu D., Genetos D.C., Shao Y., Geist D.J., Li J., Ke H.Z., Turner C.H., Duncan R.L. (2008). Activation of extracellular-signal regulated kinase (ERK1/2) by fluid shear is Ca^2+^- and ATP-dependent in MC3T3-E1 osteoblasts. Bone.

[93] Danciu T.E., Adam R.M., Naruse K., Freeman M.R., Hauschka P.V. (2003). Calcium regulates the PI3K-Akt pathway in stretched osteoblasts. FEBS Lett..

[94] Kuroda Y., Hisatsune C., Nakamura T., Matsuo K., Mikoshiba K. (2008). Osteoblasts induce Ca2+ oscillation-independent NFATc1 activation during osteoclastogenesis. Proc. Natl. Acad. Sci. USA.

[95] Julien M., Khoshniat S., Lacreusette A., Gatius M., Bozec A., Wagner E.F., Wittrant Y., Masson M., Weiss P., Beck L. (2009). Phosphate-Dependent Regulation of MGP in Osteoblasts: Role of ERK1/2 and Fra-1. J. Bone Miner. Res..

[96] Tada H., Nemoto E., Foster B.L., Somerman M.J., Shimauchi H. (2011). Phosphate increases bone morphogenetic protein-2 expression through cAMP-dependent protein kinase and ERK1/2 pathways in human dental pulp cells. Bone.

[97] Mozar A., Haren N., Chasseraud M., Louvet L., Mazière C., Wattel A., Mentaverri R., Morlière P., Kamel S., Brazier M. (2007). High extracellular inorganic phosphate concentration inhibits RANK-RANKL signaling in osteoclast-like cells. J. Cell. Physiol..

[98] Zhang R., Lu Y., Ye L., Yuan B., Yu S., Qin C., Xie Y., Gao T., Drezner M.K., Bonewald L.F. (2010). Unique roles of phosphorus in endochondral bone formation and osteocyte maturation. J. Bone Miner. Res..

[99] Lee E.J., Kasper F.K., Mikos A.G. (2014). Biomaterials for Tissue Engineering. Ann. Biomed. Eng..

[100] Laskus A., Kolmas J. (2017). Ionic Substitutions in Non-Apatitic Calcium Phosphates. Int. J. Mol. Sci..

[101] Chandran S., John A. (2018). Osseointegration of osteoporotic bone implants: Role of stem cells, Silica and Strontium—A concise review. J. Clin. Orthop. Trauma.

[102] Ni G.X., Chiu K.-Y., Lu W.W., Wang Y., Zhang Y.G., Hao L.B., Li Z.Y., Lam W.M., Lu S.B., Luk K.D.K. (2006). Strontium-containing hydroxyapatite bioactive bone cement in revision hip arthroplasty. Biomaterials.

[103] Ni G.X., Lu W.W., Chiu K.Y., Li Z.Y., Fong D.Y.T., Luk K.D.K. (2005). Strontium-containing hydroxyapatite (Sr-HA) bioactive cement for primary hip replacement: An in vivo study. J. Biomed. Mater. Res. Part B Appl. Biomater..

[104] Tian M., Chen F., Song W., Song Y., Chen Y., Wan C., Yu X., Zhang X. (2009). In vivo study of porous strontium-doped calcium polyphosphate scaffolds for bone substitute applications. J. Mater. Sci. Mater. Med..

[105] Thormann U., Ray S., Sommer U., El Khassawna T., Rehling T., Hundgeburth M., Henß A., Rohnke M., Janek J., Lips K.S. (2013). Bone formation induced by strontium modified calcium phosphate cement in critical-size metaphyseal fracture defects in ovariectomized rats. Biomaterials.

[106] Chen Y.W., Feng T., Shi G.Q., Ding Y.L., Yu X.X., Zhang X.H., Zhang Z.B., Wan C.X. (2008). Interaction of endothelial cells with biodegradable strontium-doped calcium polyphosphate for bone tissue engineering. Appl. Surf. Sci..

[107] Capuccini C., Torricelli P., Sima F., Boanini E., Ristoscu C., Bracci B., Socol G., Fini M., Mihailescu I.N., Bigi A. (2008). Strontium-substituted hydroxyapatite coatings synthesized by pulsed-laser deposition: In vitro osteoblast and osteoclast response. Acta Biomater..

[108] Yan S., Xia P., Xu S., Zhang K., Li G., Cui L., Yin J. (2018). Nanocomposite Porous Microcarriers Based on Strontium-Substituted HA-g-Poly(γ-benzyl-l-glutamate) for Bone Tissue Engineering. ACS Appl. Mater. Interfaces.

[109] Fu J., Zhuang C., Qiu J., Ke X., Yang X., Jin Z., Zhang L., Yang G., Xie L., Xu S. (2019). Core–Shell Biphasic Microspheres with Tunable Density of Shell Micropores Providing Tailorable Bone Regeneration. Tissue Eng. Part A.

[110] Liu D., Nie W., Li D., Wang W., Zheng L., Zhang J., Zhang J., Peng C., Mo X., He C. (2019). 3D printed PCL/SrHA scaffold for enhanced bone regeneration. Chem. Eng. J..

[111] Reitmaier S., Kovtun A., Schuelke J., Kanter B., Lemm M., Hoess A., Heinemann S., Nies B., Ignatius A. (2018). Strontium(II) and mechanical loading additively augment bone formation in calcium phosphate scaffolds. J. Orthop. Res..

[112] Xie H., Gu Z., He Y., Xu J., Xu C., Li L., Ye Q. (2018). Microenvironment construction of strontium–calcium-based biomaterials for bone tissue regeneration: The equilibrium effect of calcium to strontium. J. Mater. Chem. B.

[113] Salamanna F., Giavaresi G., Contartese D., Bigi A., Boanini E., Parrilli A., Lolli R., Gasbarrini A., Brodano G.B., Fini M. (2019). Effect of strontium substituted ß-TCP associated to mesenchymal stem cells from bone marrow and adipose tissue on spinal fusion in healthy and ovariectomized rat. J. Cell. Physiol..

[114] Chakar C., Naaman N., Soffer E., Cohen N., El Osta N., Petite H., Anagnostou F. (2014). Bone Formation with Deproteinized Bovine Bone Mineral or Biphasic Calcium Phosphate in the Presence of Autologous Platelet Lysate: Comparative Investigation in Rabbit. Int. J. Biomater..

[115] Aroni M.A.T., De Oliveira G.J.P.L., Spolidorio L.C., Andersen O.Z., Foss M., Marcantonio R.A.C., Stavropoulos A. (2018). Loading deproteinized bovine bone with strontium enhances bone regeneration in rat calvarial critical size defects. Clin. Oral Investig..

[116] Elgali I., Turri A., Xia W., Norlindh B., Johansson A., Dahlin C., Thomsen P., Omar O. (2016). Guided bone regeneration using resorbable membrane and different bone substitutes: Early histological and molecular events. Acta Biomater..

[117] Kaur G., Pickrell G., Sriranganathan N., Kumar V., Homa D. (2015). Review and the state of the art: Sol-gel and melt quenched bioactive glasses for tissue engineering. J. Biomed. Mater. Res. Part B Appl. Biomater..

[118] Arcos D., Vallet-Regí M. (2010). Sol–gel silica-based biomaterials and bone tissue regeneration. Acta Biomater..

[119] Vallet-Regi M., Salinas A. (2021). Mesoporous bioactive glasses for regenerative medicine. Mater. Today Bio.

[120] Kaur G., Kumar V., Baino F., Mauro J.C., Pickrell G., Evans I., Bretcanu O. (2019). Mechanical properties of bioactive glasses, ceramics, glass-ceramics and composites: State-of-the-art review and future challenges. Mater. Sci. Eng. C.

[121] Bari A., Molino G., Fiorilli S., Vitale-Brovarone C. (2018). Novel multifunctional strontium-copper co-substituted mesoporous bioactive particles. Mater. Lett..

[122] Rahaman M.N., Day D.E., Bal B.S., Fu Q., Jung S.B., Bonewald L.F., Tomsia A.P. (2011). Bioactive glass in tissue engineering. Acta Biomater..

[123] Zheng K., Niu W., Lei B., Boccaccini A.R. (2021). Immunomodulatory bioactive glasses for tissue regeneration. Acta Biomater..

[124] O’Donnell M.D., Hill R.G. (2010). Influence of strontium and the importance of glass chemistry and structure when designing bioactive glasses for bone regeneration. Acta Biomater..

[125] Zhang Y., Wei L., Chang J., Miron R.J., Shi B., Yi S., Wu C. (2013). Strontium-incorporated mesoporous bioactive glass scaffolds stimulating in vitro proliferation and differentiation of bone marrow stromal cells and in vivo regeneration of osteoporotic bone defects. J. Mater. Chem. B.

[126] Fiorilli S., Molino G., Pontremoli C., Iviglia G., Torre E., Cassinelli C., Morra M., Vitale-Brovarone C., Fiorilli S., Molino G. (2018). The Incorporation of Strontium to Improve Bone-Regeneration Ability of Mesoporous Bioactive Glasses. Materials.

[127] Pontremoli C., Izquierdo-Barba I., Montalbano G., Vallet-Regí M., Vitale-Brovarone C., Fiorilli S. (2020). Strontium-releasing mesoporous bioactive glasses with anti-adhesive zwitterionic surface as advanced biomaterials for bone tissue regeneration. J. Colloid Interface Sci..

[128] Fiorilli S., Pagani M., Boggio E., Gigliotti C.L., Dianzani C., Gauthier R., Pontremoli C., Montalbano G., Dianzani U., Vitale-Brovarone C. (2021). Sr-Containing Mesoporous Bioactive Glasses Bio-Functionalized with Recombinant ICOS-Fc: An In Vitro Study. Nanomaterials.

[129] Autefage H., Allen F., Tang H.M., Kallepitis C., Gentleman E., Reznikov N., Nitiputri K., Nommeots-Nomm A., O’Donnell M.D., Lange C. (2019). Multiscale analyses reveal native-like lamellar bone repair and near perfect bone-contact with porous strontium-loaded bioactive glass. Biomaterials.

[130] Shaltooki M., Dini G., Mehdikhani M. (2019). Fabrication of chitosan-coated porous polycaprolactone/strontium-substituted bioactive glass nanocomposite scaffold for bone tissue engineering. Mater. Sci. Eng. C.

[131] Zhang Q., Chen X., Geng S., Wei L., Miron R.J., Zhao Y., Zhang Y. (2017). Nanogel-based scaffolds fabricated for bone regeneration with mesoporous bioactive glass and strontium: In vitro and in vivo characterization. J. Biomed. Mater. Res. Part A.

[132] Poh P.S.P., Hutmacher D.W., Stevens M.M., Woodruff M.A. (2013). Fabrication and in vitro characterization of bioactive glass composite scaffolds for bone regeneration. Biofabrication.

[133] Wu C., Zhou Y., Lin C., Chang J., Xiao Y. (2012). Strontium-containing mesoporous bioactive glass scaffolds with improved osteogenic/cementogenic differentiation of periodontal ligament cells for periodontal tissue engineering. Acta Biomater..

[134] Zhang J., Zhao S., Zhu Y., Huang Y., Zhu M., Tao C., Zhang C. (2014). Three-dimensional printing of strontium-containing mesoporous bioactive glass scaffolds for bone regeneration. Acta Biomater..

[135] Ren J., Blackwood K.A., Doustgani A., Poh P.P., Steck R., Stevens M.M., Woodruff M.A. (2013). Melt-electrospun polycaprolactone strontium-substituted bioactive glass scaffolds for bone regeneration. J. Biomed. Mater. Res. Part A.

[136] Midha S., Kumar S., Sharma A., Kaur K., Shi X., Naruphontjirakul P., Jones J.R., Ghosh S. (2018). Silk fibroin-bioactive glass based advanced biomaterials: Towards patient-specific bone grafts. Biomed. Mater..

[137] Poh P.S.P., Hutmacher D.W., Holzapfel B.M., Solanki A.K., Stevens M.M., Woodruff M.A. (2016). In vitro and in vivo bone formation potential of surface calcium phosphate-coated polycaprolactone and polycaprolactone/bioactive glass composite scaffolds. Acta Biomater..

[138] Baheiraei N., Eyni H., Bakhshi B., Najafloo R., Rabiee N. (2021). Effects of strontium ions with potential antibacterial activity on in vivo bone regeneration. Sci. Rep..

[139] Kargozar S., Montazerian M., Fiume E., Baino F. (2019). Multiple and Promising Applications of Strontium (Sr)-Containing Bioactive Glasses in Bone Tissue Engineering. Front. Bioeng. Biotechnol..

[140] Cui X., Huang C., Zhang M., Ruan C., Peng S., Li L., Liu W., Wang T., Li B., Huang W. (2017). Enhanced osteointegration of poly(methylmethacrylate) bone cements by incorporating strontium-containing borate bioactive glass. J. R. Soc. Interface.

[141] Fernandes J.S., Gentile P., Martins M., Neves N.M., Miller C., Crawford A., Pires R.A., Hatton P., Reis R.L. (2016). Reinforcement of poly-l-lactic acid electrospun membranes with strontium borosilicate bioactive glasses for bone tissue engineering. Acta Biomater..

[142] Fernandes J.S., Gentile P., Crawford A., Pires R.A., Hatton P.V., Reis R.L. (2017). Substituted Borosilicate Glasses with Improved Osteogenic Capacity for Bone Tissue Engineering. Tissue Eng. Part A.

[143] Brow R.K. (2000). Review: The structure of simple phosphate glasses. J. Non-Cryst. Solids.

[144] Lakhkar N.J., Lee I.-H., Kim H.-W., Salih V., Wall I.B., Knowles J.C. (2013). Bone formation controlled by biologically relevant inorganic ions: Role and controlled delivery from phosphate-based glasses. Adv. Drug Deliv. Rev..

[145] Patel U., Macri-Pellizzeri L., Hossain K.M.Z., Scammell B.E., Grant D.M., Scotchford C.A., Hannon A.C., Kennedy A.R., Barney E.R., Ahmed I. (2019). In vitro cellular testing of strontium/calcium substituted phosphate glass discs and microspheres shows potential for bone regeneration. J. Tissue Eng. Regen. Med..

[146] Hanawa T. (2019). Titanium–Tissue Interface Reaction and Its Control with Surface Treatment. Front. Bioeng. Biotechnol..

[147] Simon M., Lagneau C., Moreno J., Lissac M., Dalard F., Grosgogeat B. (2005). Corrosion resistance and biocompatibility of a new porous surface for titanium implants. Eur. J. Oral Sci..

[148] Xin Y., Jiang J., Huo K., Hu T., Chu P.K. (2009). Bioactive SrTiO3 Nanotube Arrays: Strontium Delivery Platform on Ti-Based Osteoporotic Bone Implants. ACS Nano.

[149] Mi B., Xiong W., Xu N., Guan H., Fang Z., Liao H., Zhang Y., Gao B., Xiao X., Fu J. (2017). Strontium-loaded titania nanotube arrays repress osteoclast differentiation through multiple signalling pathways: In vitro and in vivo studies. Sci. Rep..

[150] Mumith A., Cheong V.S., Fromme P., Coathup M.J., Blunn G.W. (2020). The effect of strontium and silicon substituted hydroxyapatite electrochemical coatings on bone ingrowth and osseointegration of selective laser sintered porous metal implants. PLoS ONE.

[151] Okuzu Y., Fujibayashi S., Yamaguchi S., Yamamoto K., Shimizu T., Sono T., Goto K., Otsuki B., Matsushita T., Kokubo T. (2017). Strontium and magnesium ions released from bioactive titanium metal promote early bone bonding in a rabbit implant model. Acta Biomater..

[152] Lee C.-H., Kim Y.-J., Jang J.-H., Park J.-W. (2016). Modulating macrophage polarization with divalent cations in nanostructured titanium implant surfaces. Nanotechnology.

[153] Kim H.-S., Kim Y.-J., Jang J.-H., Park J.-W. (2016). Surface Engineering of Nanostructured Titanium Implants with Bioactive Ions. J. Dent. Res..

[154] Zhou J., Wang X., Zhao L. (2019). Antibacterial, angiogenic, and osteogenic activities of Ca, P, Co, F, and Sr compound doped titania coatings with different Sr content. Sci. Rep..

[155] Offermanns V., Andersen O.Z., Riede G., Sillassen M., Jeppesen C.S., Almtoft K.P., Talasz H., Öhman-Mägi C., Lethaus B., Tolba R. (2018). Effect of strontium surface-functionalized implants on early and late osseointegration: A histological, spectrometric and tomographic evaluation. Acta Biomater..

[156] Wang H., Xu Q., Hu H., Shi C., Lin Z., Jiang H., Dong H., Guo J. (2020). The Fabrication and Function of Strontium-modified Hierarchical Micro/Nano Titanium Implant. Int. J. Nanomed..

[157] Alenezi A., Galli S., Atefyekta S., Andersson M., Wennerberg A. (2019). Osseointegration effects of local release of strontium ranelate from implant surfaces in rats. J. Mater. Sci. Mater. Med..

[158] Li D., Li Y., Shrestha A., Wang S., Wu Q., Li L., Guan C., Wang C., Fu T., Liu W. (2019). Effects of Programmed Local Delivery from a Micro/Nano-Hierarchical Surface on Titanium Implant on Infection Clearance and Osteogenic Induction in an Infected Bone Defect. Adv. Healthc. Mater..

[159] van Hengel I.A.J., Gelderman F.S.A., Athanasiadis S., Minneboo M., Weinans H., Fluit A.C., van der Eerden B.C.J., Fratila-Apachitei L.E., Apachitei I., Zadpoor A.A. (2020). Functionality-packed additively manufactured porous titanium implants. Mater. Today Bio.

[160] Cheng H., Xiong W., Fang Z., Guan H., Wu W., Li Y., Zhang Y., Alvarez M.M., Gao B., Huo K. (2016). Strontium (Sr) and silver (Ag) loaded nanotubular structures with combined osteoinductive and antimicrobial activities. Acta Biomater..

[161] Filippi M., Born G., Chaaban M., Scherberich A. (2020). Natural Polymeric Scaffolds in Bone Regeneration. Front. Bioeng. Biotechnol..

[162] Bonani W., Singhatanadgige W., Pornanong A., Motta A. (2018). Natural Origin Materials for Osteochondral Tissue Engineering. Advances in Experimental Medicine and Biology.

[163] Rao S.H., Harini B., Shadamarshan R.P.K., Balagangadharan K., Selvamurugan N. (2018). Natural and synthetic polymers/bioceramics/bioactive compounds-mediated cell signalling in bone tissue engineering. Int. J. Biol. Macromol..

[164] Lee S.-H., Shin H. (2007). Matrices and scaffolds for delivery of bioactive molecules in bone and cartilage tissue engineering. Adv. Drug Deliv. Rev..

[165] Lei B., Guo B., Rambhia K.J., Ma P.X. (2018). Hybrid polymer biomaterials for bone tissue regeneration. Front. Med..

[166] Chen Y., Zheng Z., Zhou R., Zhang H., Chen C., Xiong Z., Liu K., Wang X. (2019). Developing a Strontium-Releasing Graphene Oxide-/Collagen-Based Organic–Inorganic Nanobiocomposite for Large Bone Defect Regeneration via MAPK Signaling Pathway. ACS Appl. Mater. Interfaces.

[167] Montalbano G., Borciani G., Pontremoli C., Ciapetti G., Mattioli-Belmonte M., Fiorilli S., Vitale-Brovarone C. (2019). Development and Biocompatibility of Collagen-Based Composites Enriched with Nanoparticles of Strontium Containing Mesoporous Glass. Materials.

[168] Montalbano G., Borciani G., Cerqueni G., Licini C., Banche-Niclot F., Janner D., Sola S., Fiorilli S., Mattioli-Belmonte M., Ciapetti G. (2020). Collagen Hybrid Formulations for the 3D Printing of Nanostructured Bone Scaffolds: An Optimized Genipin-Crosslinking Strategy. Nanomaterials.

[169] Borciani G., Montalbano G., Melo P., Baldini N., Ciapetti G., Vitale-Brovarone C. (2021). Assessment of Collagen-Based Nanostructured Biomimetic Systems with a Co-Culture of Human Bone-Derived Cells. Cells.

[170] Fenbo M., Xingyu X., Bin T. (2019). Strontium chondroitin sulfate/silk fibroin blend membrane containing microporous structure modulates macrophage responses for guided bone regeneration. Carbohydr. Polym..

[171] Lu S., Wang P., Zhang F., Zhou X., Zuo B., You X., Gao Y., Liu H., Tang H. (2015). A novel silk fibroin nanofibrous membrane for guided bone regeneration: A study in rat calvarial defects. Am. J. Transl. Res..

[172] Luz E.P.C.G., Borges M.D.F., Andrade F.K., Rosa M.D.F., Infantes-Molina A., Rodríguez-Castellón E., Vieira R.S. (2018). Strontium delivery systems based on bacterial cellulose and hydroxyapatite for guided bone regeneration. Cellulose.

[173] Cheng D., Liang Q., Li Y., Fan J., Wang G., Pan H., Ruan C. (2018). Strontium incorporation improves the bone-forming ability of scaffolds derived from porcine bone. Colloids Surfaces B Biointerfaces.

[174] Lino A.B., McCarthy A.D., Fernández J.M. (2019). Evaluation of Strontium-Containing PCL-PDIPF Scaffolds for Bone Tissue Engineering: In Vitro and In Vivo Studies. Ann. Biomed. Eng..

[175] Prabha R.D., Nair B.P., Ditzel N., Kjems J., Nair P.D., Kassem M. (2019). Strontium functionalized scaffold for bone tissue engineering. Mater. Sci. Eng. C.

[176] Jiménez M., Abradelo C., Román J.S., Rojo L. (2019). Bibliographic review on the state of the art of strontium and zinc based regenerative therapies. Recent developments and clinical applications. J. Mater. Chem. B.

[177] Wei P., Jing W., Yuan Z., Huang Y., Guan B., Zhang W., Zhang X., Mao J., Cai Q., Chen D. (2019). Vancomycin- and Strontium-Loaded Microspheres with Multifunctional Activities against Bacteria, in Angiogenesis, and in Osteogenesis for Enhancing Infected Bone Regeneration. ACS Appl. Mater. Interfaces.

[178] Rodríguez-Méndez I., Fernández-Gutiérrez M., Rodríguez-Navarrete A., Rosales-Ibañez R., Benito-Garzón L., Vázquez-Lasa B., Román J.S. (2018). Bioactive Sr(II)/Chitosan/Poly(ε-caprolactone) Scaffolds for Craniofacial Tissue Regeneration. In Vitro and In Vivo Behavior. Polymers.

[179] Wang X., Shao J., El Raouf M.A., Xie H., Huang H., Wang H., Chu P., Yu X.-F., Yang Y., AbdEl-Aal A.M. (2018). Near-infrared light-triggered drug delivery system based on black phosphorus for in vivo bone regeneration. Biomaterials.

[180] Loca D., Smirnova A., Locs J., Dubnika A., Vecstaudza J., Stipniece L., Makarova E., Dambrova M. (2018). Development of local strontium ranelate delivery systems and long term in vitro drug release studies in osteogenic medium. Sci. Rep..

[181] O’Neill E., Awale G., Daneshmandi L., Umerah O., Lo K.W.-H. (2018). The roles of ions on bone regeneration. Drug Discov. Today.

[182] Blair H.C., Larrouture Q.C., Li Y., Lin H., Beer-Stoltz D., Liu L., Tuan R.S., Robinson L.J., Schlesinger P.H., Nelson D.J. (2017). Osteoblast Differentiation and Bone Matrix Formation In Vivo and In Vitro. Tissue Eng. Part B Rev..

[183] Girón J., Kerstner E., Medeiros T., Oliveira L., Machado G.M., Malfatti C.F., Pranke P. (2021). Biomaterials for bone regeneration: An orthopedic and dentistry overview. Braz. J. Med. Biol. Res..

[184] Naruphontjirakul P., Tsigkou O., Li S., Porter A.E., Jones J.R. (2019). Human mesenchymal stem cells differentiate into an osteogenic lineage in presence of strontium containing bioactive glass nanoparticles. Acta Biomater..

